# Supplementary material for: Two-thirds of global cropland area impacted by climate oscillations
Source: Nat Commun. 2018 Mar 28;9:1257. doi: 10.1038/s41467-017-02071-5 (PMC5871885; doi:10.1038/s41467-017-02071-5)
Supplement: Supplementary file 1 — Supplementary Information(PDF 5599 kb) [file 41467_2017_2071_MOESM1_ESM.pdf]

Supplementary Note 1: Brief introduction to ENSO, IOD and NAO.

ENSO (El-Niño Southern Oscillation) is a pattern of sea-surface temperature (SST) and pressure gradients between the eastern and western parts of the equatorial Pacific Ocean[1]. It is the dominant source of interannual climate variability around the globe. When SST is abnormally high (low) in the eastern Pacific Ocean and the pressure gradient between eastern and western Pacific Ocean is low (high), the state of ENSO is here positive (El Niño), while the negative phase represents La Niña. During an El Niño event, the westerlies in the equatorial Pacific Ocean weaken, which hinders the uprising of cold water, leading to warm SST anomalies along the coasts of Peru and Ecuador. El Niño also results in shifts in the equatorial large-scale evaporation, condensation and wind patterns, causing climate anomalies around the globe through so-called teleconnections[2].

IOD (Indian Ocean Dipole) refers to the occurrence of opposite SST anomalies in the western Indian Ocean and on the coasts of northern Indonesia[3]. Although some connection between ENSO and IOD exists, only 20 to 25% of IOD variability can be explained by ENSO, and a notable proportion of IOD events occur independent of ENSO[4] (and vice versa). During the evolution of a positive (negative) IOD, cold (warm) SST anomalies appear in the eastern part of the Indian Ocean, with warm (cold) anomalies in the western part. These SST anomalies change the patterns of zonal winds, moisture transport and precipitation in the neighboring areas as well as elsewhere.

NAO (North Atlantic Oscillation) is defined as the difference in atmospheric pressure between northern (Iceland) and southern (Azores) parts of the North Atlantic Ocean[5]. When the pressure gradient from the southern high-pressure to the northern low-pressure region is high (low), NAO is in its positive (negative) phase. The positive phase of NAO strengthens westerlies in the northern hemisphere, which modulates the climate from western Europe all the way to China[6].

Supplementary Note 2: Climatological effects of ENSO, IOD and NAO.

The climatological patterns related to ENSO events are widely studied. At global scale, El Niño has been found to be associated with lower precipitation in Southeast Asia, northern South America, northeastern Australia[7] as well as parts of southern[8] and northern[9] Africa. These are all areas where this study found significant relationships between ENSO and crop productivity. Temperature variability in these areas also shows relationships with ENSO. For example, a global study conducted by Trenberth & Caron[8] found significant negative correlations between the Southern Oscillation Index (gives opposite values compared to the index used in this study) and land surface temperatures (LST) in Southeast Asia, northern South America, southern and eastern Africa as well as South Asia. Also, the Palmer Drought Severity Index (PDSI) patterns related to ENSO follow the results of this study remarkably well[10].

A global study conducted by Saji & Yamagata[4] found strong positive partial (impact of ENSO filtered) correlations between LST and IOD in southern Australia, Japan, the Korean peninsula and southern Africa. Hence, positive IOD events tend to increase LST in these areas, which are also represented in the results of this study. Further, the same study revealed that a positive phase in the IOD tends to be related with higher precipitation in central Africa and northern India, while it is related with lower precipitation in eastern Australia and South Africa. Studies conducted at sub-global level were also inspected, and especially in Australia, the effects of the positive phase have been extensively linked to high temperatures[11], low precipitation[12-14] and extreme drought events[12]. Furthermore, higher precipitation has been found to follow the positive IOD phase in southern[15, 16] and central Africa[13, 16] as well as northern India[13, 17]. However, the relationship between Indian precipitation and IOD has been found to be non-linear and related to the phase of ENSO[18].

To the best of our knowledge, no global study exists of NAO's impacts on global hydroclimatological patterns. However, several regional and local studies do exist. Hurrell et al.[6] showed a relationship between the positive phase of NAO and higher temperatures in large parts of the northern hemisphere, from western Europe to China and North America. For West Europe, these patterns are further confirmed by regional studies, which show that winter temperatures tend to be higher during a positive NAO phase, especially in France, Germany, United Kingdom as well as Scandinavia and several parts of East Europe[19]. For the Middle East, studies show an opposite effect, as a positive phase in NAO tends to be associated with below average temperatures[19-21]. Furthermore, a positive NAO phase tends to be associated with lower precipitation in Mediterranean Europe and the Middle East[21, 22] as well as some parts of northern Africa[23], and higher precipitation in several areas in the northern Europe[22]. Furthermore, summer NAO has been found to influence weather patterns even in parts of India and Central Asia[24].

Supplementary Note 3: Temporal changes in the relationship between crop productivity and oscillation indices.

We decided to examine potential changes in the linkages between teleconnections and crop productivity, because it is well documented in the literature that the strength, magnitude and activity of the studied climate oscillations vary both temporally and spatially[5, 25-28]. This variability is especially well documented for ENSO, as its power has changed over time on timescales from millennia to decades[29-32]. Moreover, many studies have shown that its influences on climates in distant regions have changed[28, 33, 34]. Ward et al.[35] showed that there has also been a change in the correlation between ENSO and flood peak discharges over the last half century.

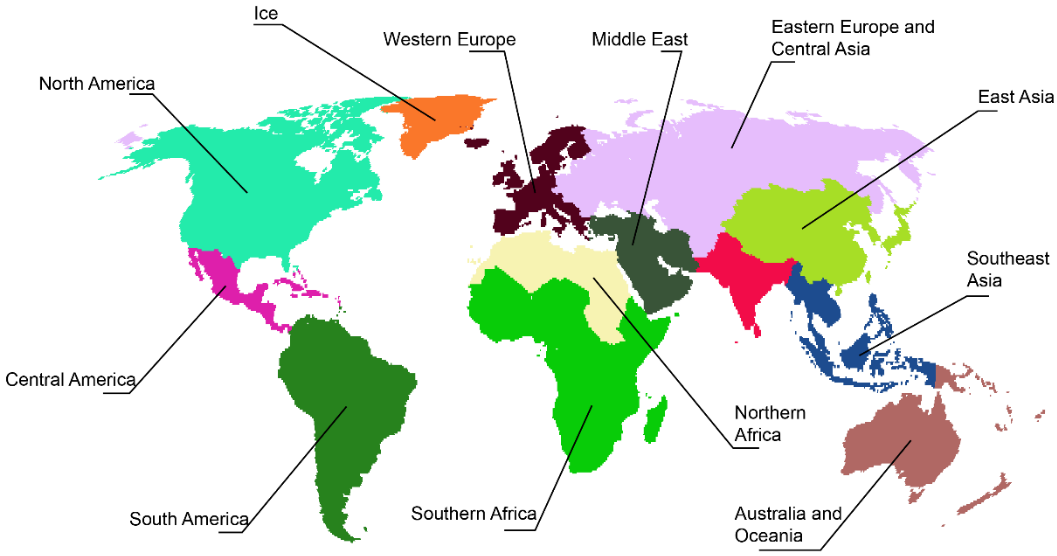

Supplementary Figure 1: Regional division used in the regional analyses of this study.

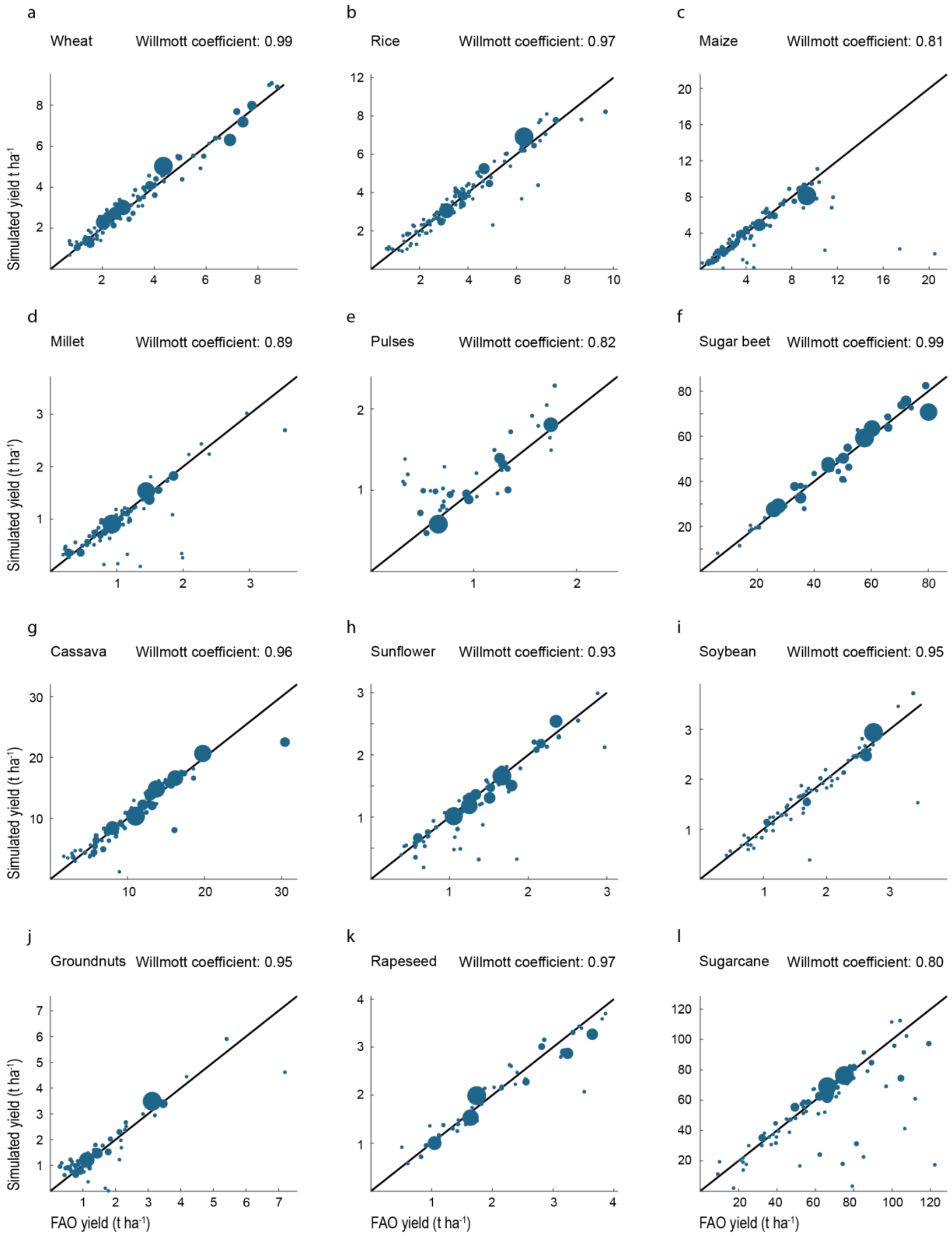

Supplementary Figure 2. Country level calibration results for all crop types included in the LPJmL model, averaged over years 2001–2010. The x-axis shows the yield according to FAOSTAT[36] *and the y-axis shows simulated yield. For each crop, the Willmott coefficient*[37] *is calculated to represent the goodness of the calibration, similarly to Fader et al.*[38] *The point sizes represent the average production for the country and time-period in question.*

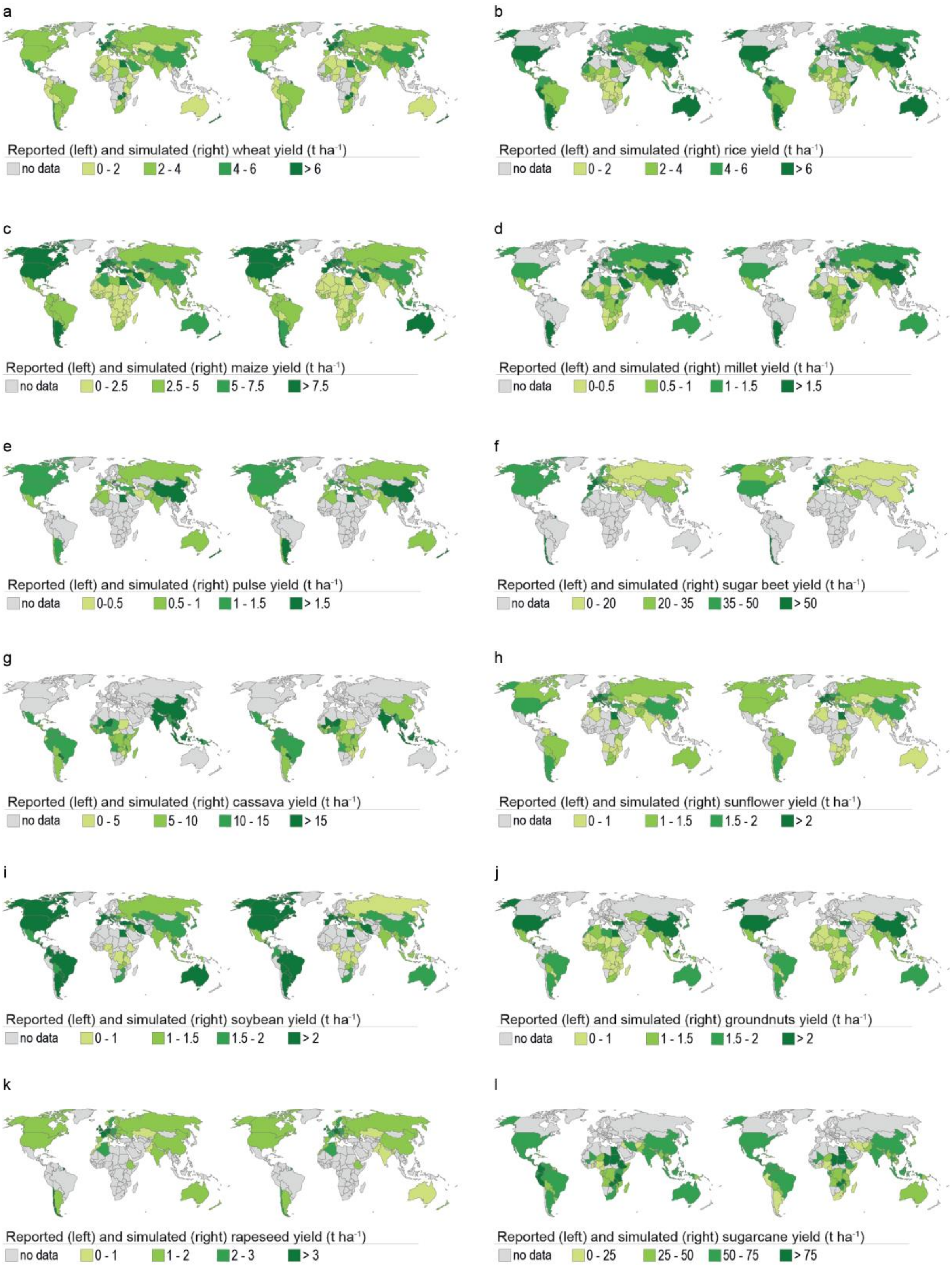

Supplementary Figure 3. Reported (FAOSTAT[36]) and simulated (calibrated) yield of all crop types included in the LPJmL model, averaged over years 2001–2010.

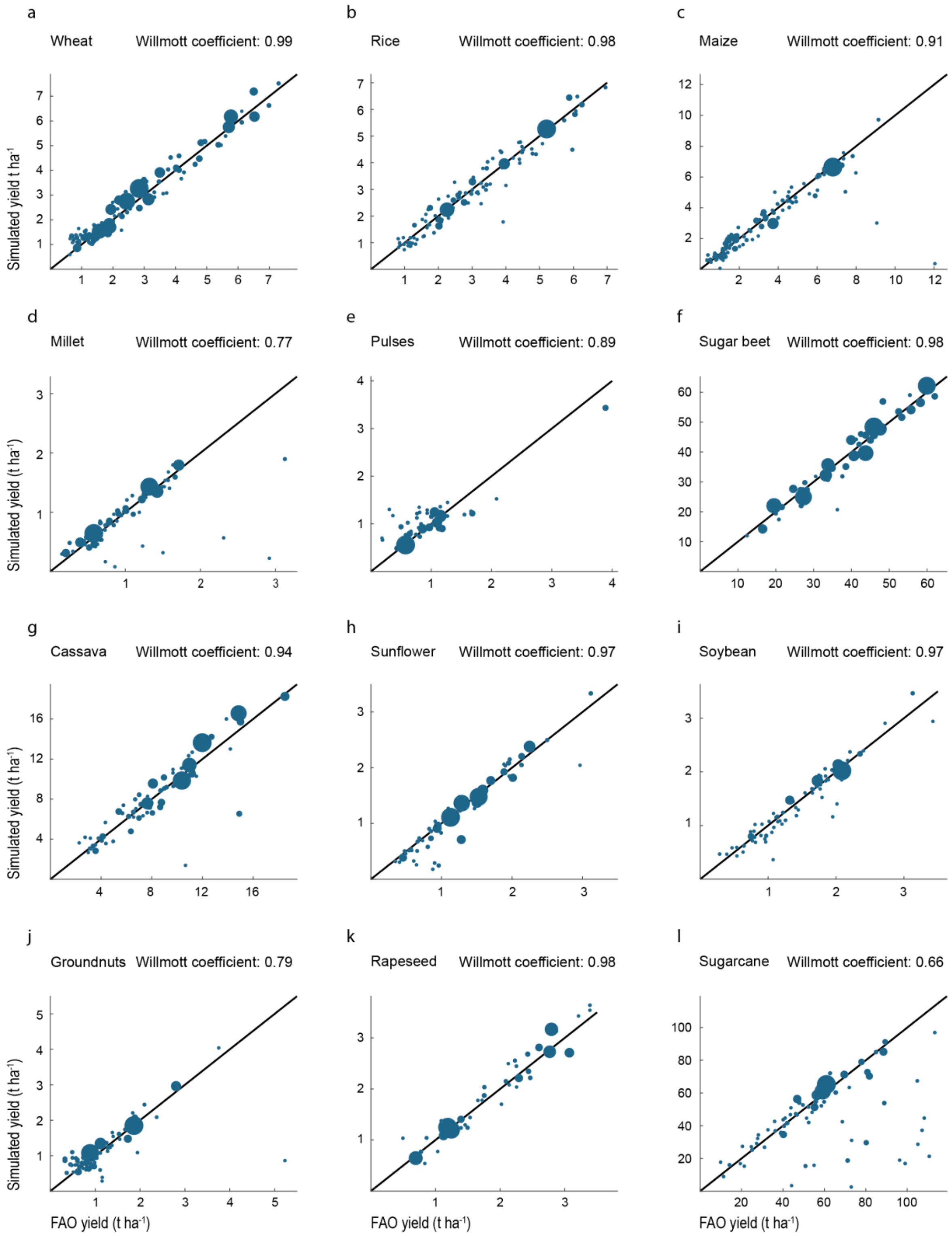

Supplementary Figure 4: Country level calibration results for all crop types included in the LPJmL model, averaged over years 1981–1990. The x-axis shows the yield according to FAOSTAT[36] and the y-axis shows simulated yield. For each crop, the Willmott coefficient[37] is calculated to represent the goodness of the calibration, similarly to Fader et al.[38] The point sizes represent the average production for the country and time-period in question.

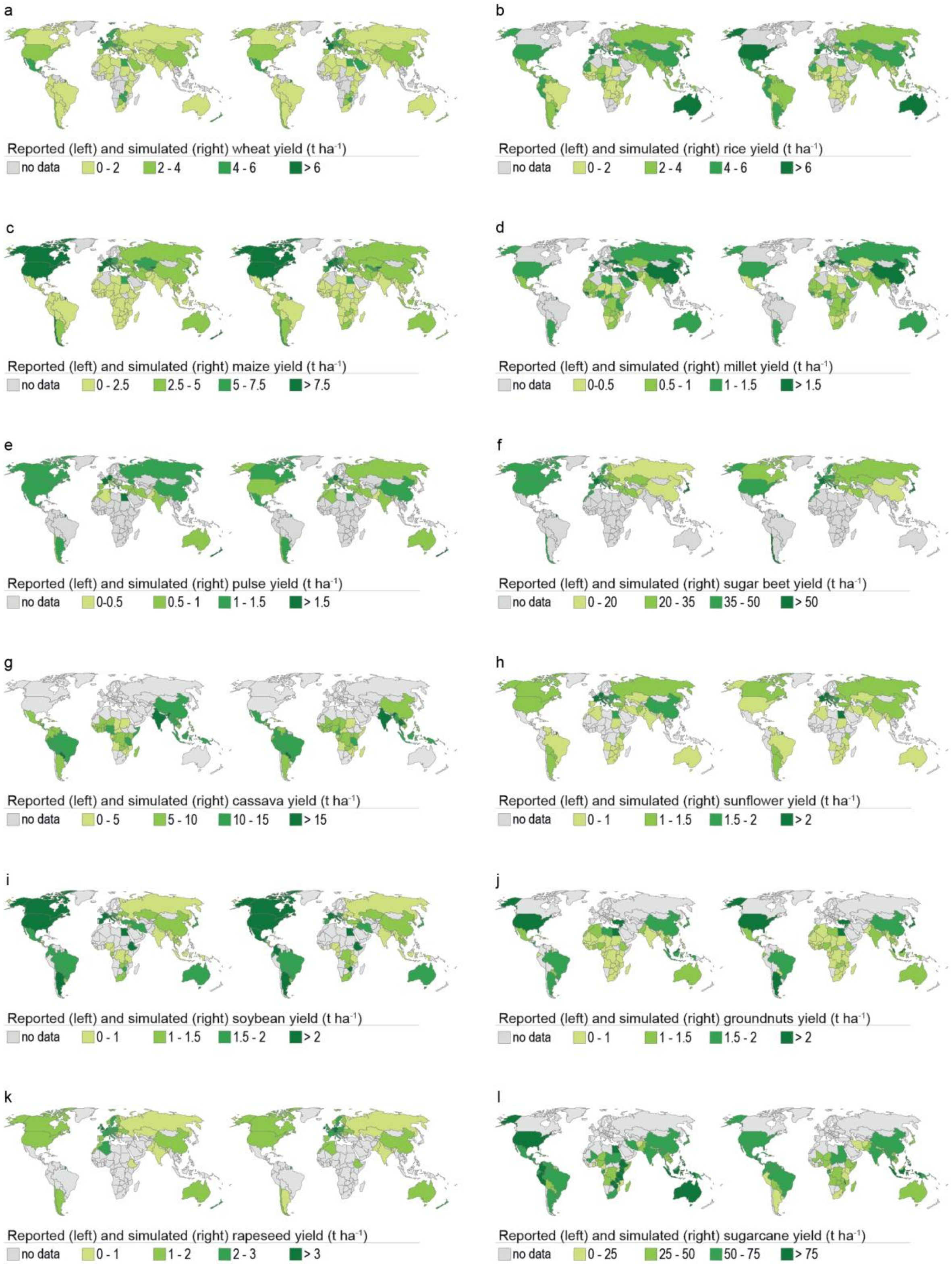

Supplementary Figure 5: Reported (FAOSTAT[36]) and simulated (calibrated) yield of all crop types included in the LPJmL model, averaged over years 1981–1990.

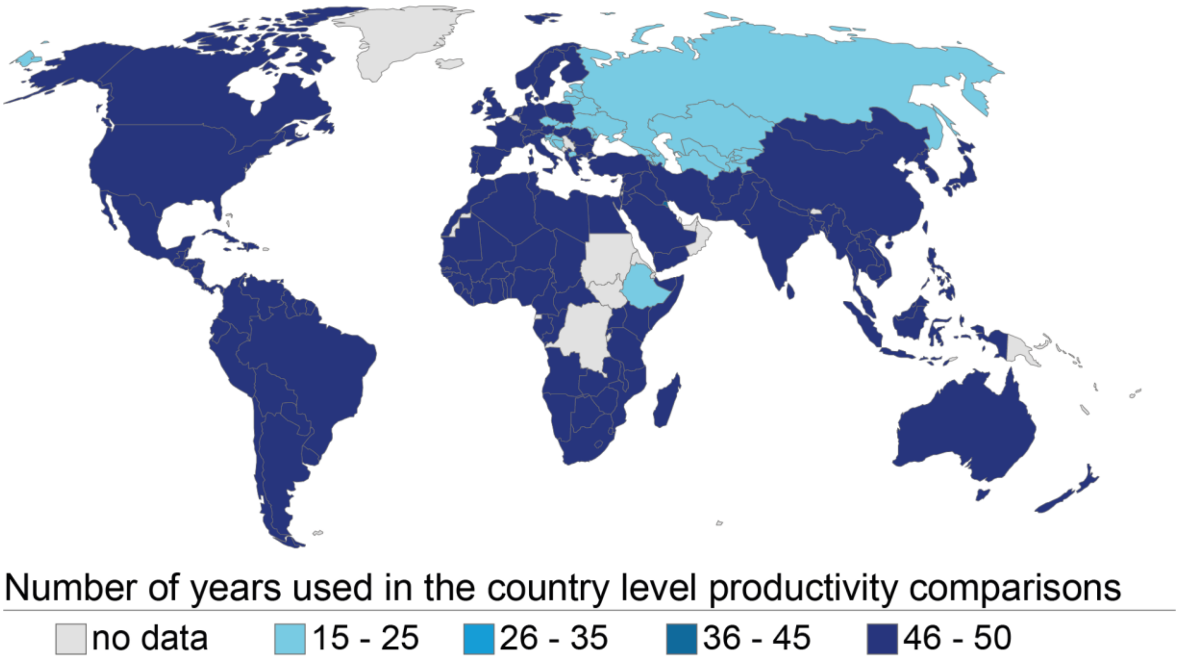

Supplementary Figure 6: Years of available productivity data from FAOSTAT[36].

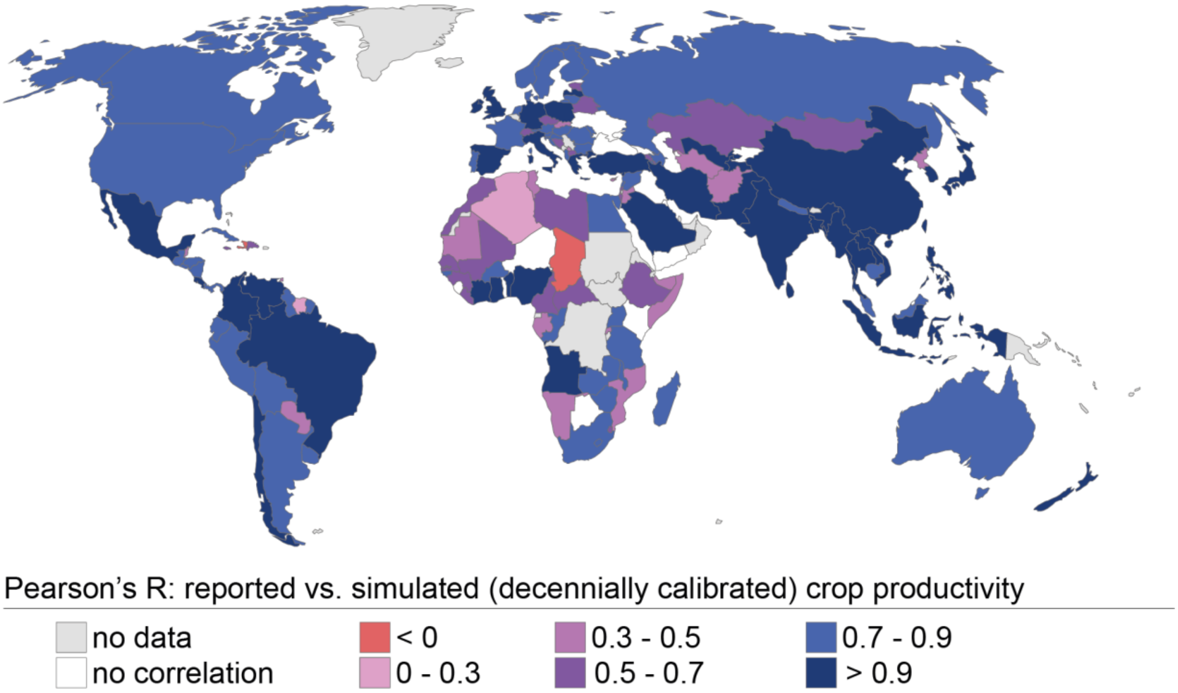

Supplementary Figure 7: Pearson's correlation between simulated (decennially calibrated) and reported (FAOSTAT[36]) crop productivity of all 12 crop types included in the LPJmL model at country scale. The first year of the correlation analysis varies depending on available data (most countries have data for the whole time-period, i.e. starting from 1961), while the last year is 2010 for all countries (see Supplementary Figure 6 for details regarding the extent of the time-periods).

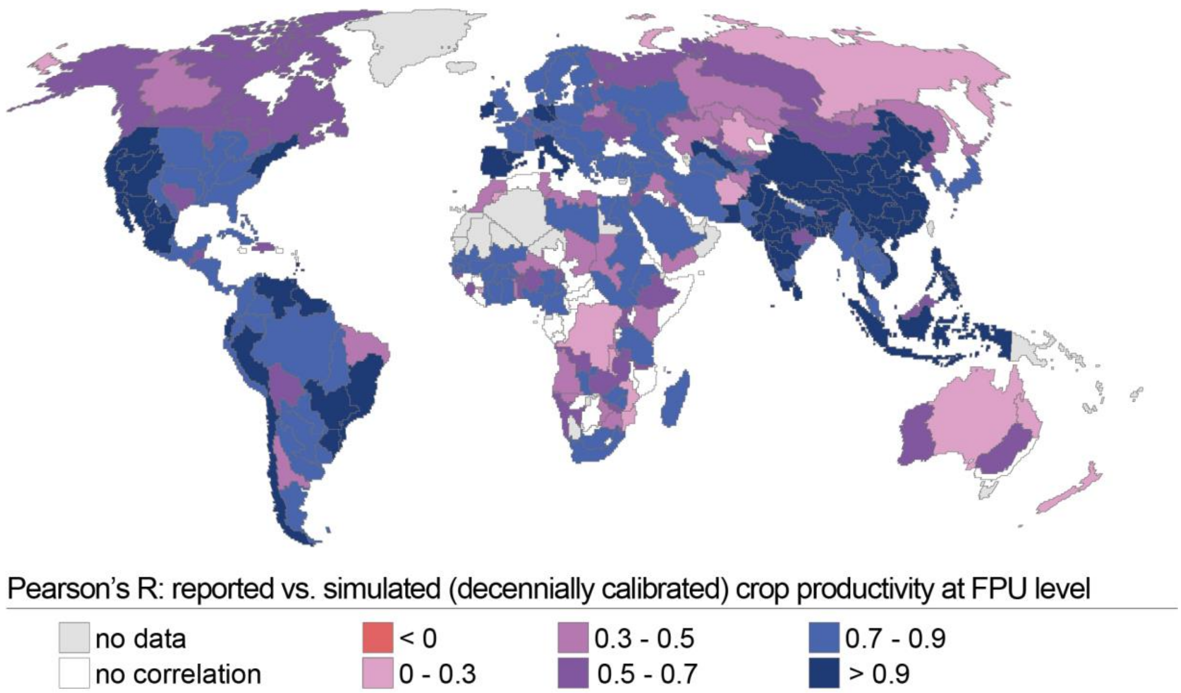

Supplementary Figure 8: Pearson's correlation between simulated (decennially calibrated) and reported crop productivity of maize, rice, soybean and wheat (Ray et al.[39]) at the FPU level for 1961-2008.

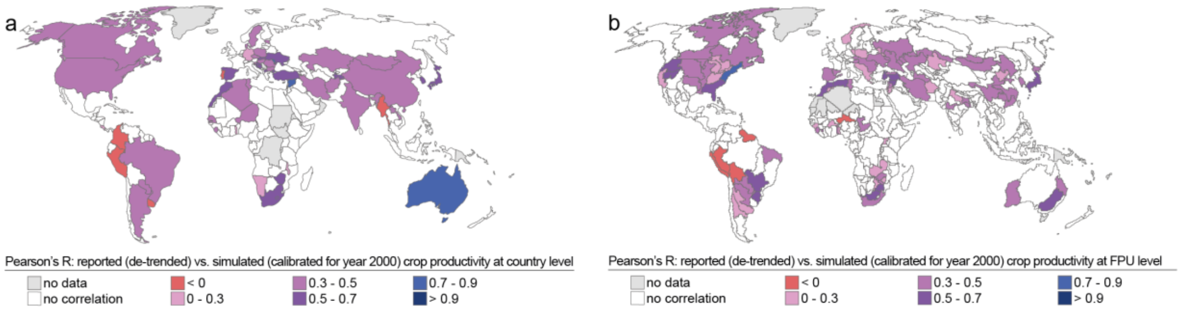

Supplementary Figure 9: Pearson's correlation between simulated (calibrated for year 2000) and reported (de-trended) crop productivity of a) the 12 crop types included in the LPJmL model at country scale (FAOSTAT[36]) as well as b) maize, rice, soybean and wheat at FPU scale (Ray et al.[39]). The de-trended reported crop productivity data was obtained by fitting and subtracting a best-fit polynomial curve from the original data. For the country scale data, the first year of the correlation analysis varies depending on available data (most countries have data for the whole time-period, i.e. starting from 1961), while the last year is 2010 for all countries (see Supplementary Figure 6 for details regarding the extent of the time-periods). For the FPU scale data the correlations were calculated for years 1961-2008. Note that, as a third of global crop yield variability can be attributed to climate variability[39] and that LPJmL is here driven by climatological input data only, it is not to be expected that it reproduces all observed crop yield dynamics.

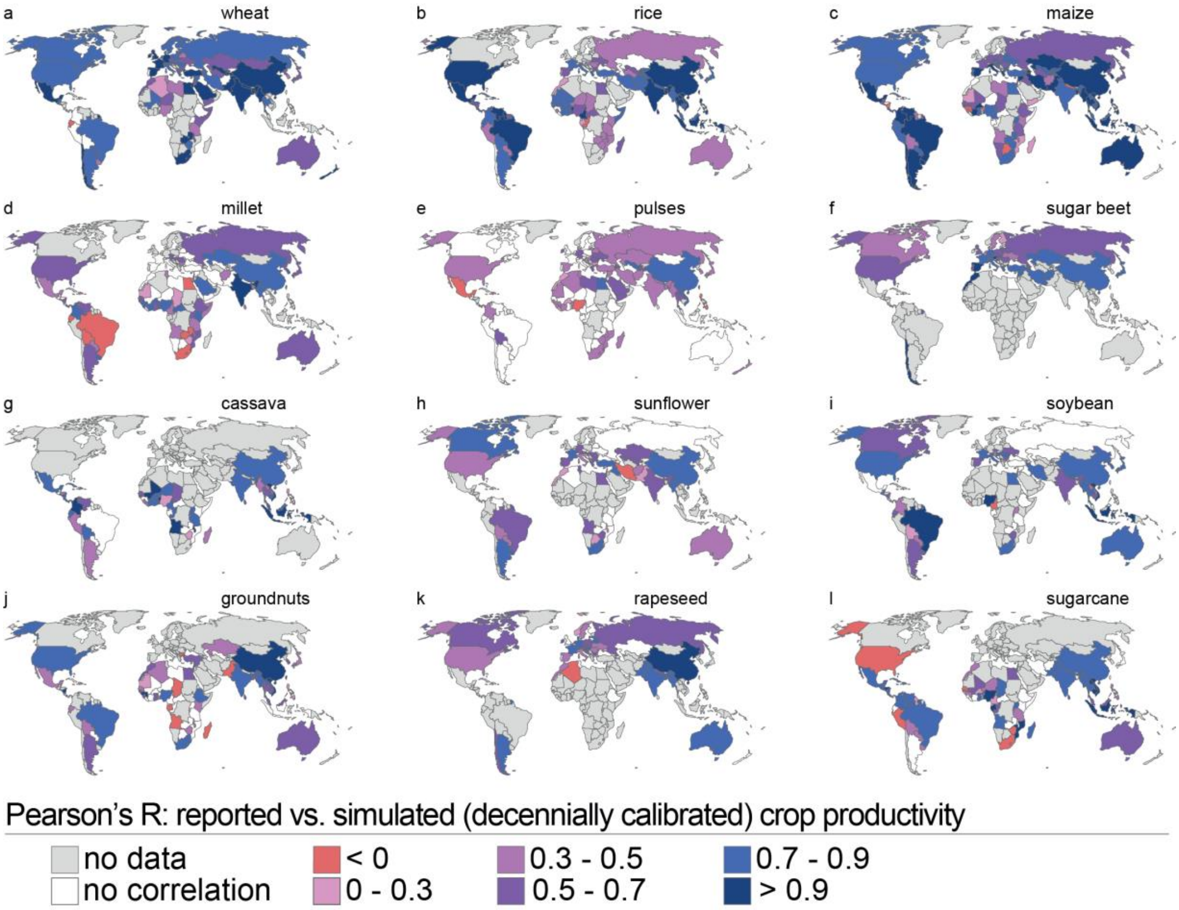

Supplementary Figure 10: Pearson's correlation between simulated (decennially calibrated) and reported crop productivity calculated for each crop type included in the LPJmL model at country scale (FAOSTAT[36]). The first year of the correlation analysis varies depending on available data (most countries have data for the whole time-period, i.e. starting from 1961), while the last year is 2010 for all countries.

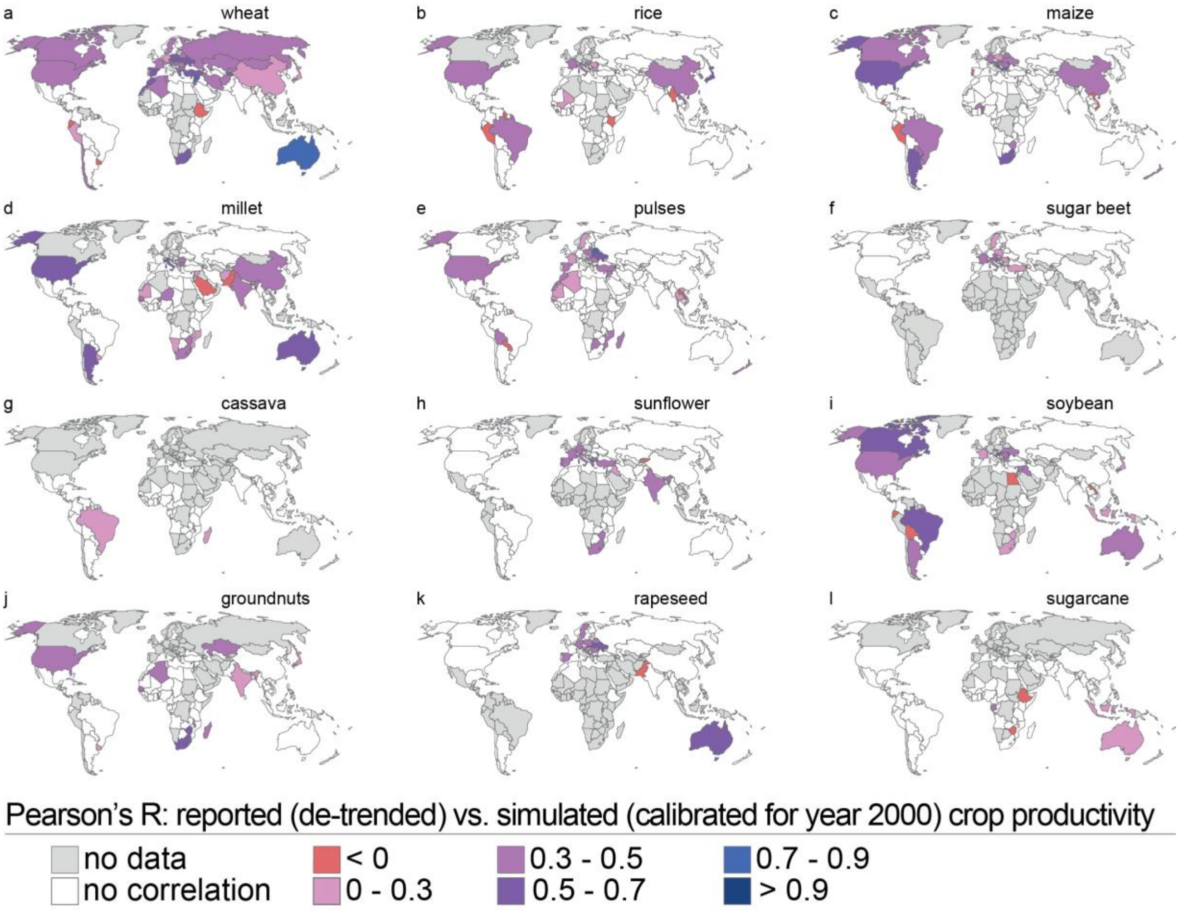

Supplementary Figure 11: Pearson's correlation between simulated (calibrated for year 2000) and reported (de-trended) crop productivity calculated for each crop type included in the LPJmL model (FAOSTAT[36]). The de-trended reported crop productivity data was obtained by fitting and subtracting a best-fit polynomial curve from the original data. The first year of the correlation analysis varies depending on available data (most countries have data for the whole time-period, i.e. starting from 1961), while the last year is 2010 for all countries.

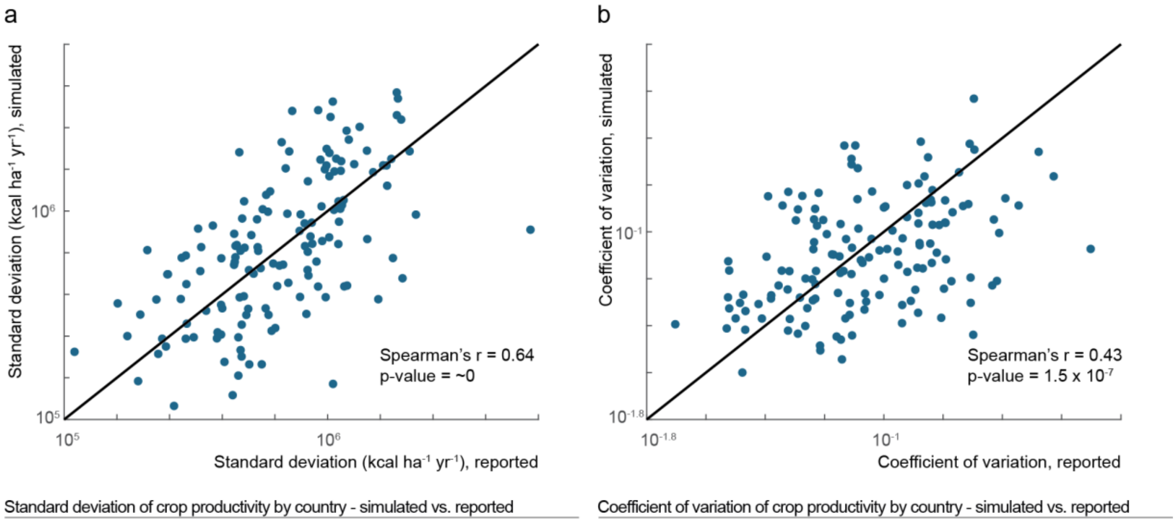

Supplementary Figure 12: a) standard deviation and b) coefficient of variation of simulated and reported crop productivity of all 12 major crop types included in the LPJmL model (FAOSTAT[36]) at the country scale for the whole study period. Prior to the variability assessment, the reported crop productivity data was de-trended by fitting and subtracting a best-fit polynomial curve from the original data. Furthermore, the average crop productivity of the reported data for calculating the CV was obtained by taking an average of the original data for the years 1995-2005. The first year of analysis varies depending on available data (most countries have data for the whole time-period, i.e. starting from 1961), while the last year is 2010 for all countries (see Supplementary Figure 6 for details regarding the extent of the time-periods).

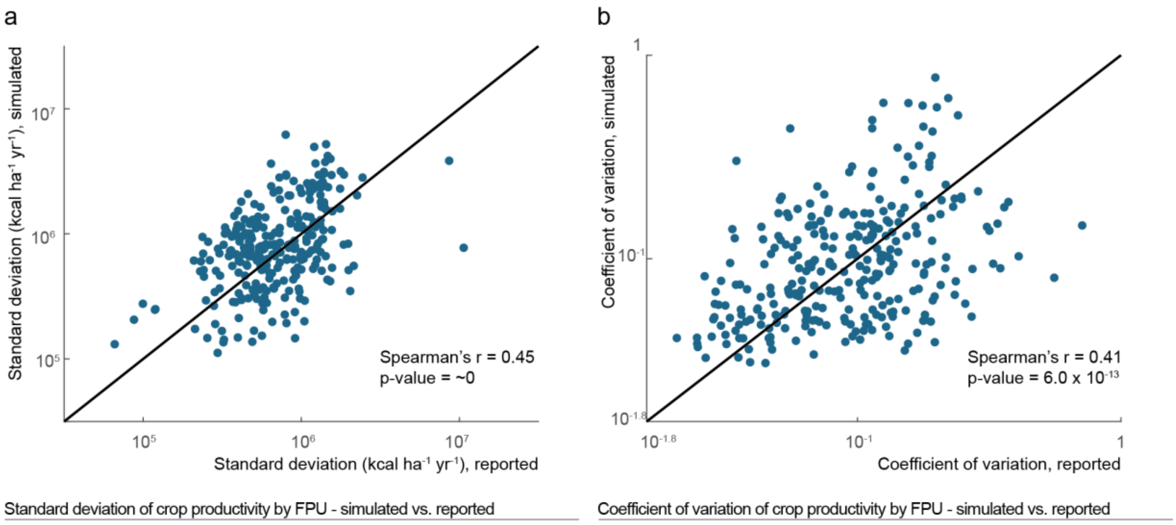

Supplementary Figure 13: a) standard deviation and b) coefficient of variation of simulated and reported crop productivity of maize, rice, soybean and wheat (Ray et al. [39]) at the FPU scale for 1961-2008. Prior to the variability assessment, the reported crop productivity data was de-trended by fitting and subtracting a best-fit polynomial curve from the original data. Furthermore, the average crop productivity of the reported data for calculating the CV was obtained by taking an average of the original data for the years 1995–2005.

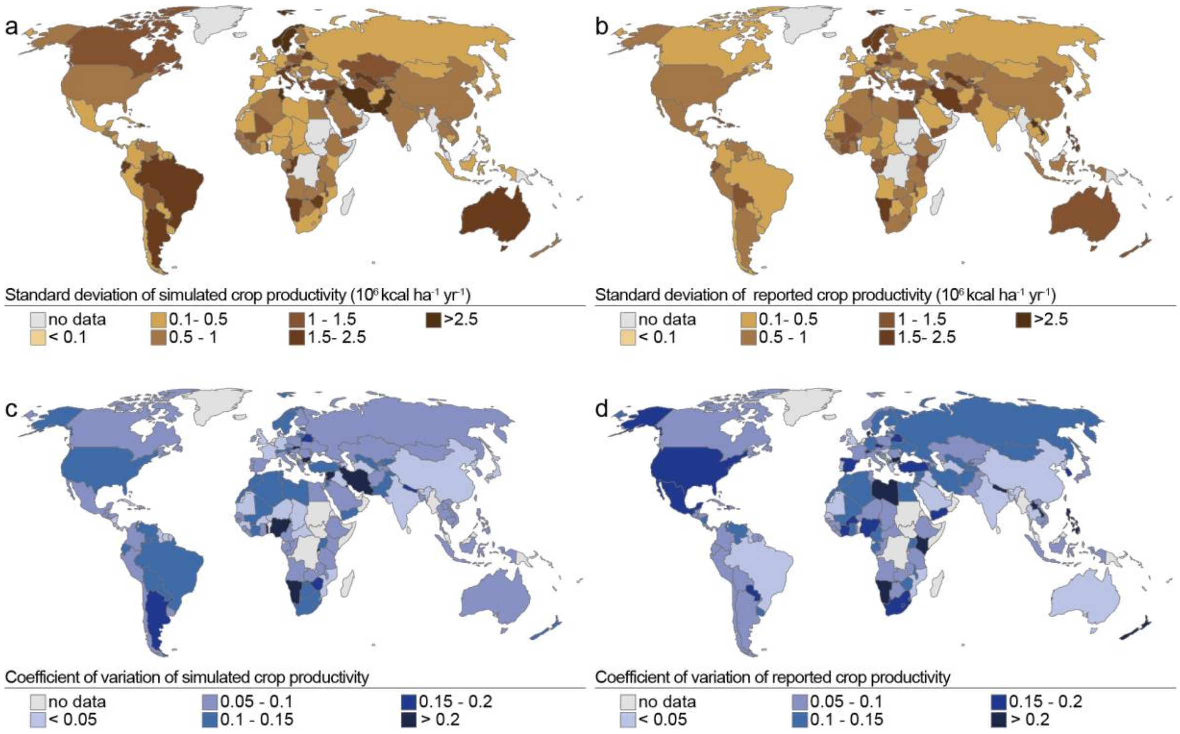

Supplementary Figure 14: Standard deviation (a & b) and coefficient of variation (c & d) of simulated (a & c) and de-trended reported (FAOSTAT[36]; b & d) crop productivity of all 12 major crop types included in the LPJmL model at the country scale. Prior to the variability assessment, the reported crop productivity data was de-trended by fitting and subtracting a best-fit polynomial curve from the original data. Furthermore, the average crop productivity for calculating the CV was obtained by taking an average of the original data for the years 1995–2005. The first year of analysis varies depending on available data (most countries have data for the whole time-period, i.e. starting from 1961), while the last year is 2010 for all countries (see Supplementary Figure 6 for details regarding the extent of the time-periods).

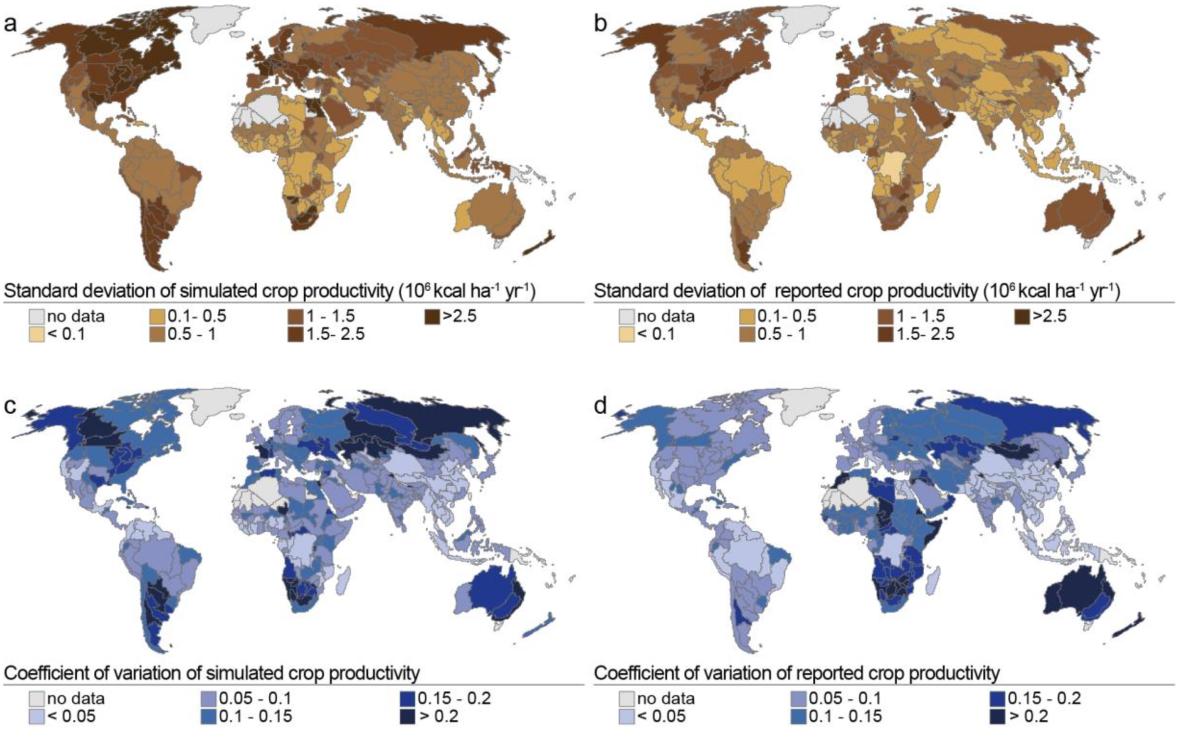

Supplementary Figure 15: Standard deviation (a & b) and coefficient of variation (c & d) of simulated (a & c) and de-trended reported (Ray et al.[39]; b & d) crop productivity of maize, rice, soybean and wheat at the FPU scale for 1961-2008. Prior to the variability assessment, the reported crop productivity data was de-trended by fitting and subtracting a best-fit polynomial curve from the original data. Furthermore, the average crop productivity of the reported data for calculating the CV was obtained by taking an average of the original data for the years 1995-2005.

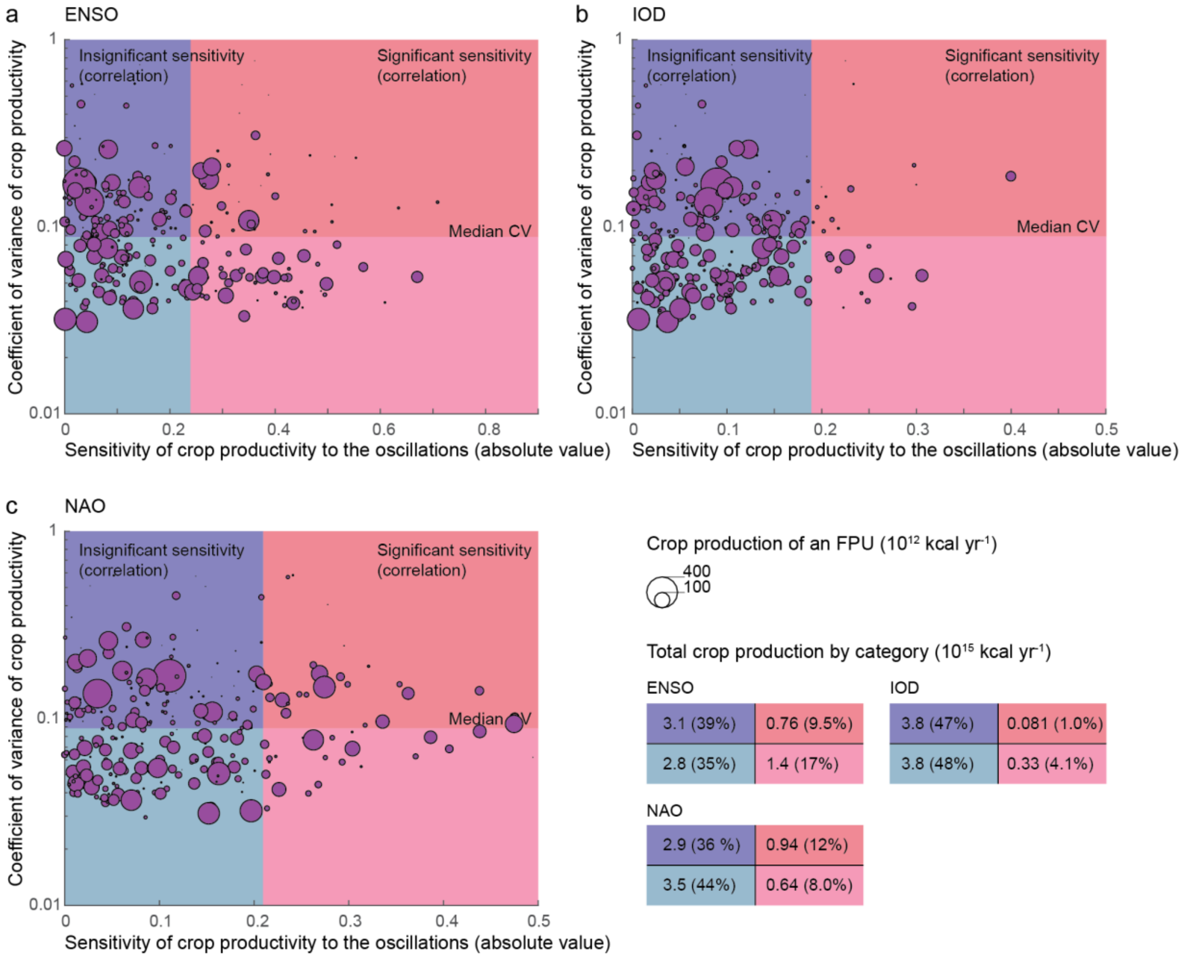

Supplementary Figure 16: FPUs mapped according to the sensitivity (absolute value) of crop productivity to the oscillation indices (x-axis) and the coefficient of variance of crop productivity (CV; y-axis) for a) ENSO, b) IOD and c) NAO. Each of the images is divided, so that in the red (blue) section the sensitivity between crop productivity and the oscillation is significant (insignificant). For CV, the figures are divided in half, so that in the upper (lower) sections of each image CV is above (below) the mean CV of all FPUs.

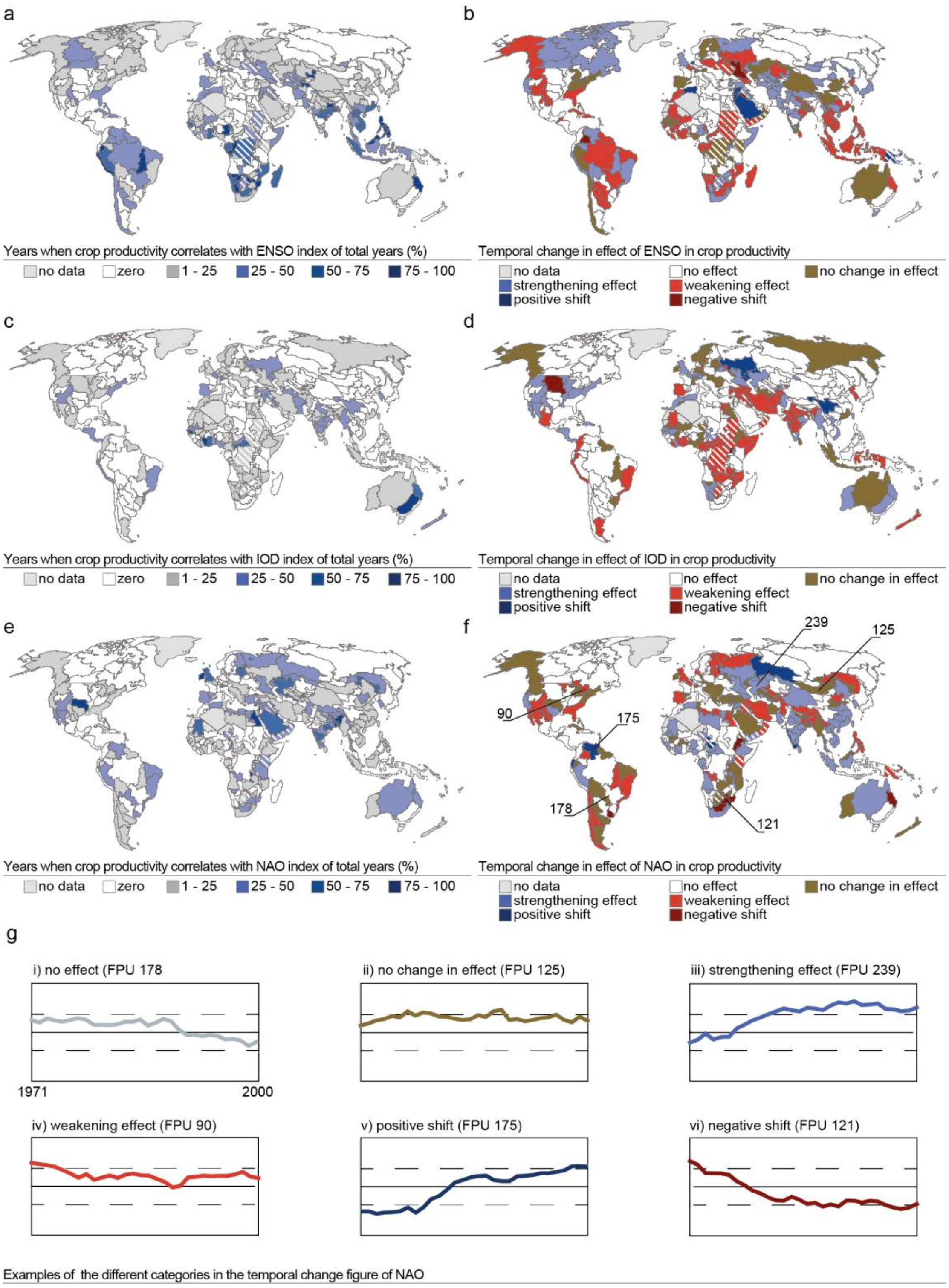

Supplementary Figure 17: Spatial and temporal variability in the relationship between the oscillations and crop productivity. a), c) & e) show the portion of years (21 year moving windows) when the oscillations have a significant (p-value < 0.1) Spearman's correlation with crop productivity, whereas b), d) and f) show whether the strength of the correlation experience changes. The bottom row (g) shows correlation time series examples of the different categories in b), d) and f). Dashed line represents the threshold for significant correlation (p < 0.1).

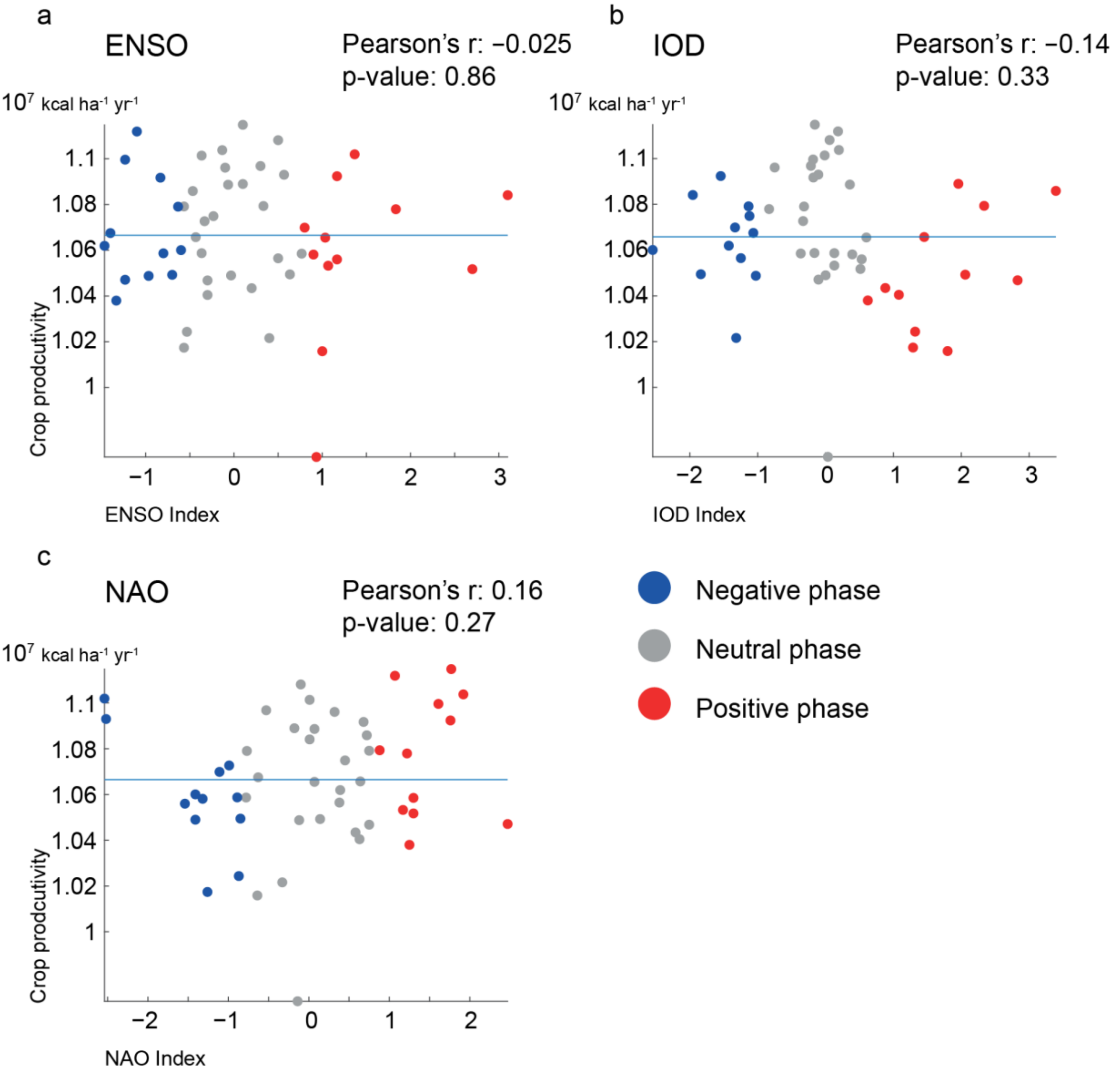

Supplementary Figure 18: Relationship between global average crop productivity of the 12 major crop types and a) ENSO, b) IOD and c) NAO indices for years 1961-2010. Years representing negative (positive) oscillation phases are marked blue (red). The blue horizontal line represents mean crop productivity for the whole time-period. In the aggregation process, the crop-specific annual yields (kg ha^{-1} yr^{-1}) of the 12 major crop types considered were converted to caloric crop productivities (kcal ha^{-1} yr^{-1}), after which the total crop productivity was calculated as the growing area weighted mean. Details about the kcal kg^{-1} conversion factors of the 12 crop types considered and the aggregation calculations at global scale for simulated and reported (FAOSTAT[36]) data can be found in Supplementary Table 10.

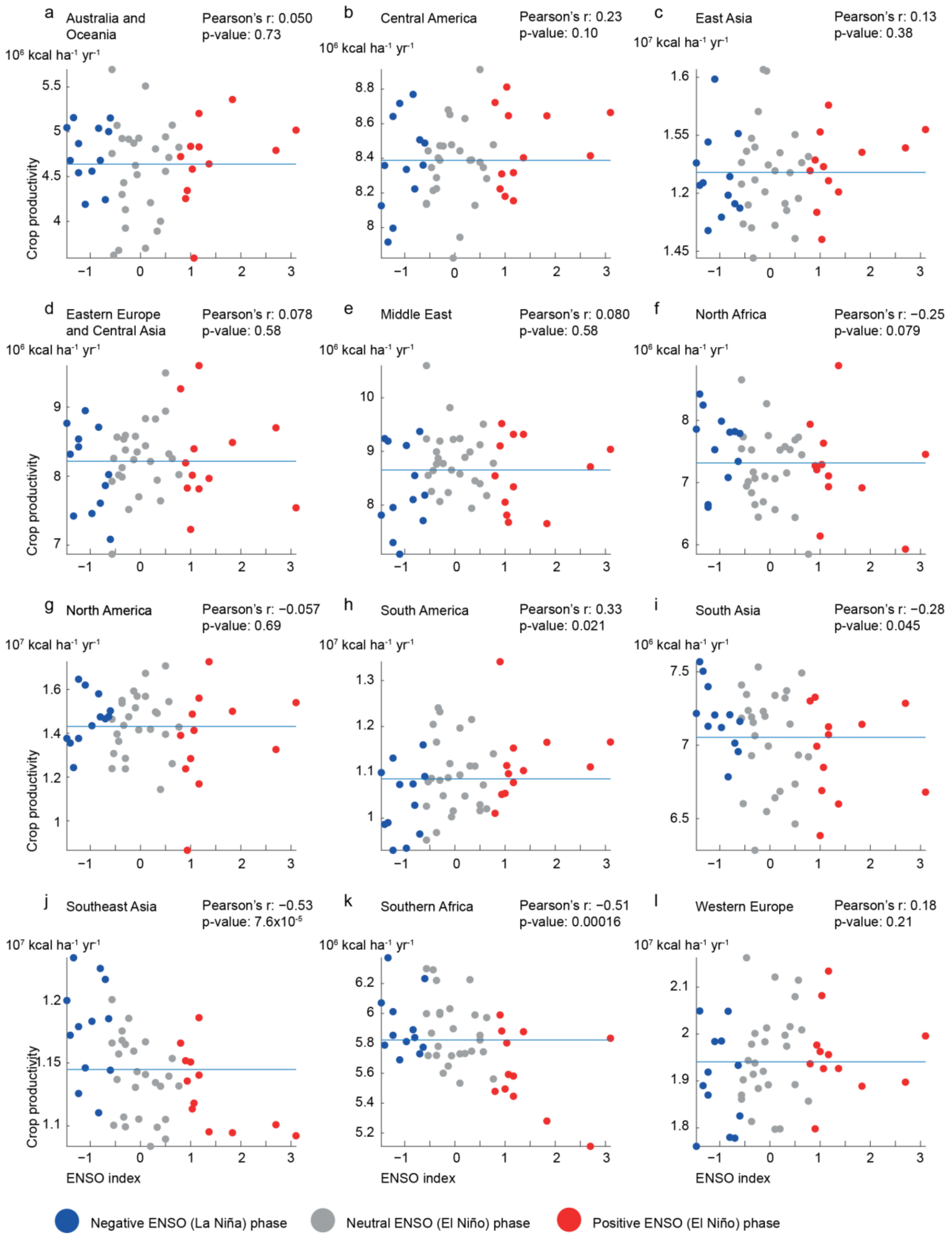

Supplementary Figure 19: Relationship between global average crop productivity of the 12 major crop types and the ENSO (JMA SST) index at regional level mapped for years 1961-2010. Years representing negative (positive) phases of ENSO are marked blue (red). The blue horizontal line represents mean crop productivity for the whole time-period. See caption of Supplementary Figure 18 and Supplementary Table 10 for details about how the crop-specific annual yields (kg ha^{-1} yr^{-1}) of the 12 major crop types considered were converted to caloric crop productivities (kcal ha^{-1} yr^{-1}).

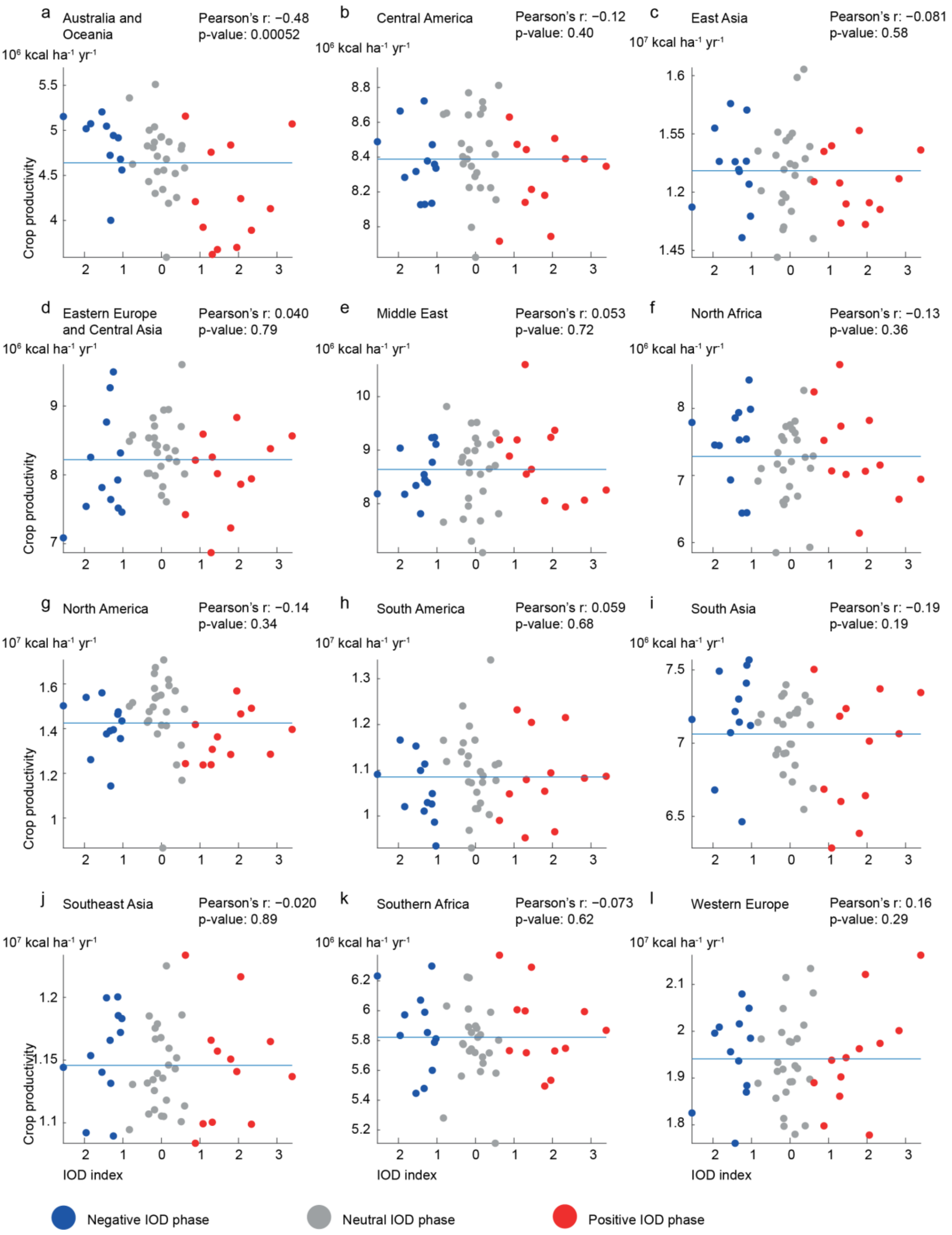

Supplementary Figure 20: Relationship between global average crop productivity of the 12 major crop types and the IOD (SST DMI) index at regional level mapped for years 1961-2010. Years representing negative (positive) phases of IOD are marked blue (red). The blue horizontal line represents mean crop productivity for the whole time-period. See caption of Supplementary Figure 18 and Supplementary Table 10 for details about how the crop-specific annual yields (kg ha^{-1} yr^{-1}) of the 12 major crop types considered were converted to caloric crop productivities (kcal ha^{-1} yr^{-1}).

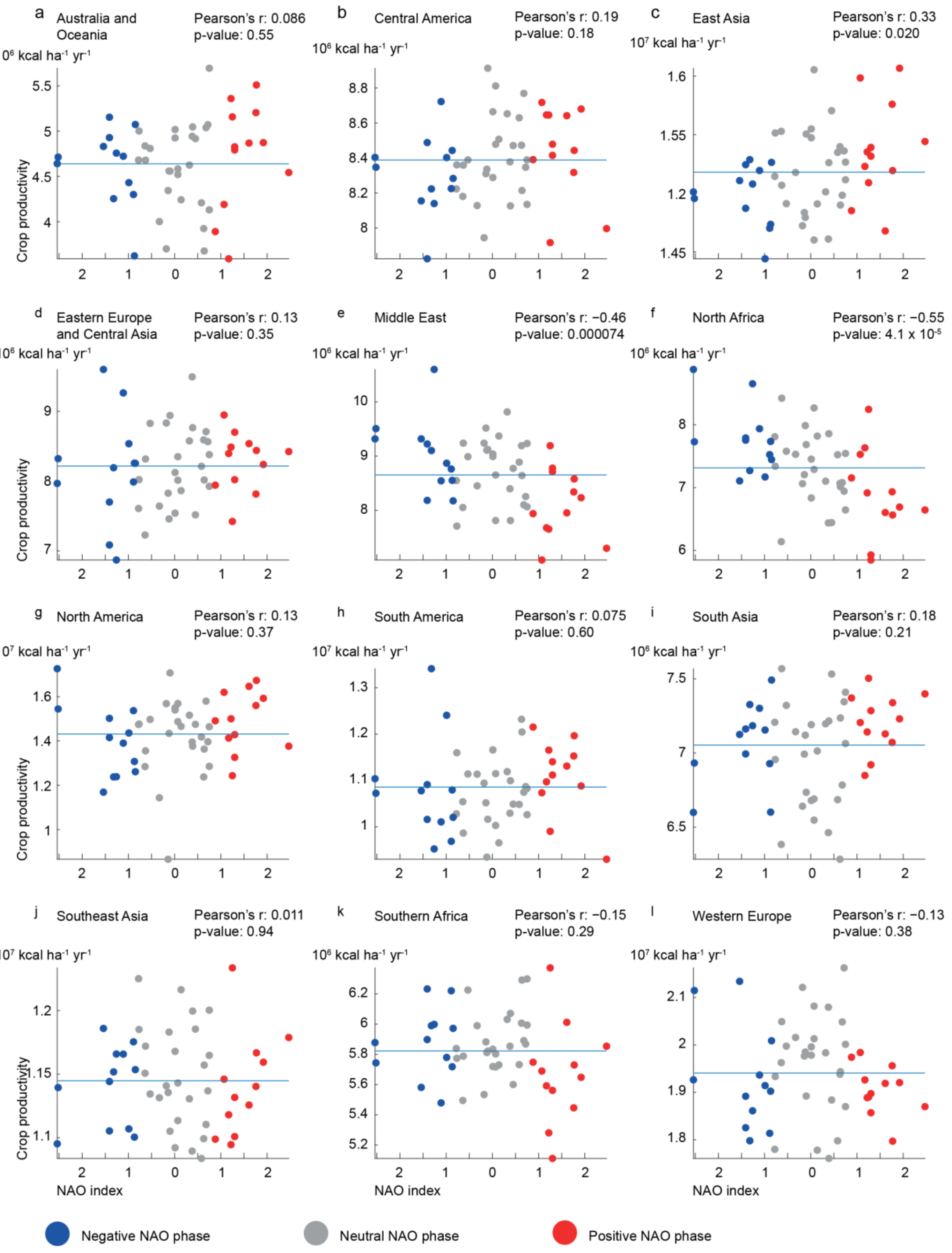

Supplementary Figure 21: Relationship between global average crop productivity of the 12 major crop types and the NAO (Hurrel North Atlantic Oscillation Index (PC-based)) index mapped for years 1961-2010. Years representing negative (positive) phases of NAO are marked blue (red). The blue horizontal line represents mean crop productivity for the whole time-period. See caption of Supplementary Figure 18 and Supplementary Table 10 for details about how the crop-specific annual yields (kg ha^{-1} yr^{-1}) of the 12 major crop types considered were converted to caloric crop productivities (kcal ha^{-1} yr^{-1}).

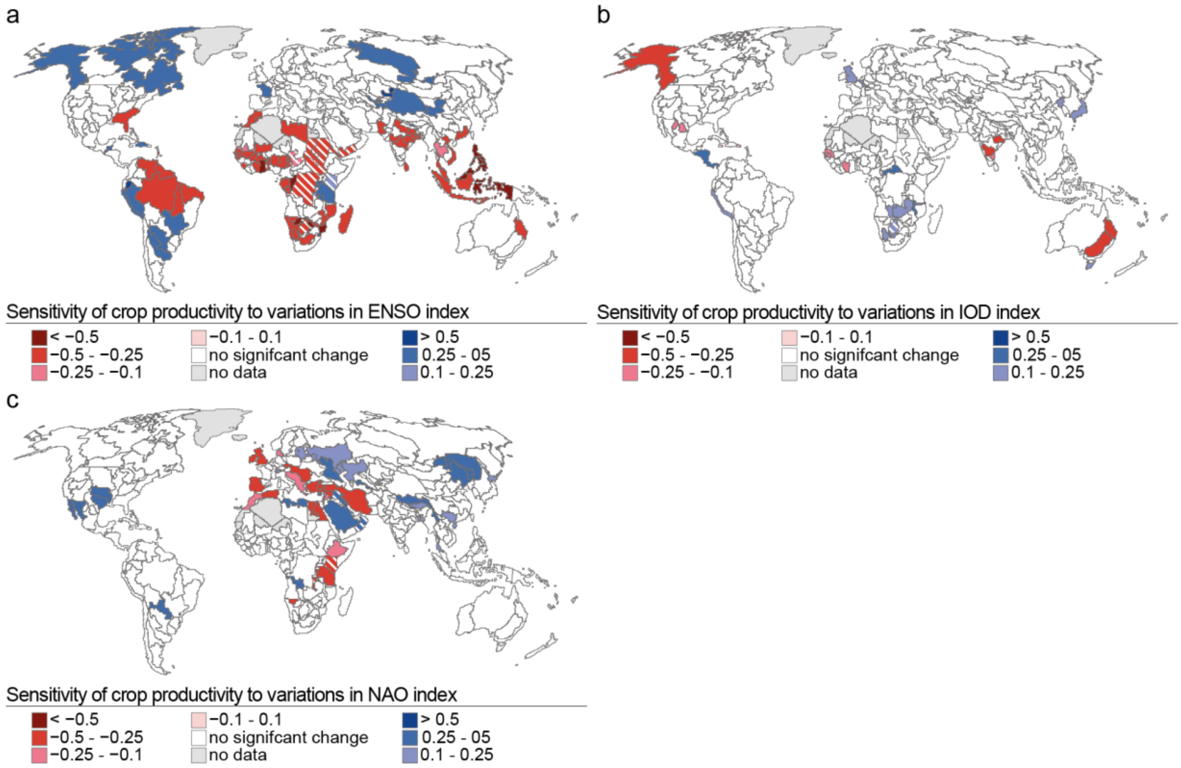

Suppelementary Figure 22: Sensitivity of crop productivity (as derived from LPJmL model with yields calibrated every 10 years, see Methods) to changes in the a) ENSO, b) IOD and c) NAO indices at FPU level. Prior to the sensitivity assessment, the crop productivity time series was de-trended by fitting and subtracting a best-fit polynomial curve from the original data. The linear relationship was concluded to be significant based on the p-value (< 0.1, parametrized) of Pearson's correlation coefficient.

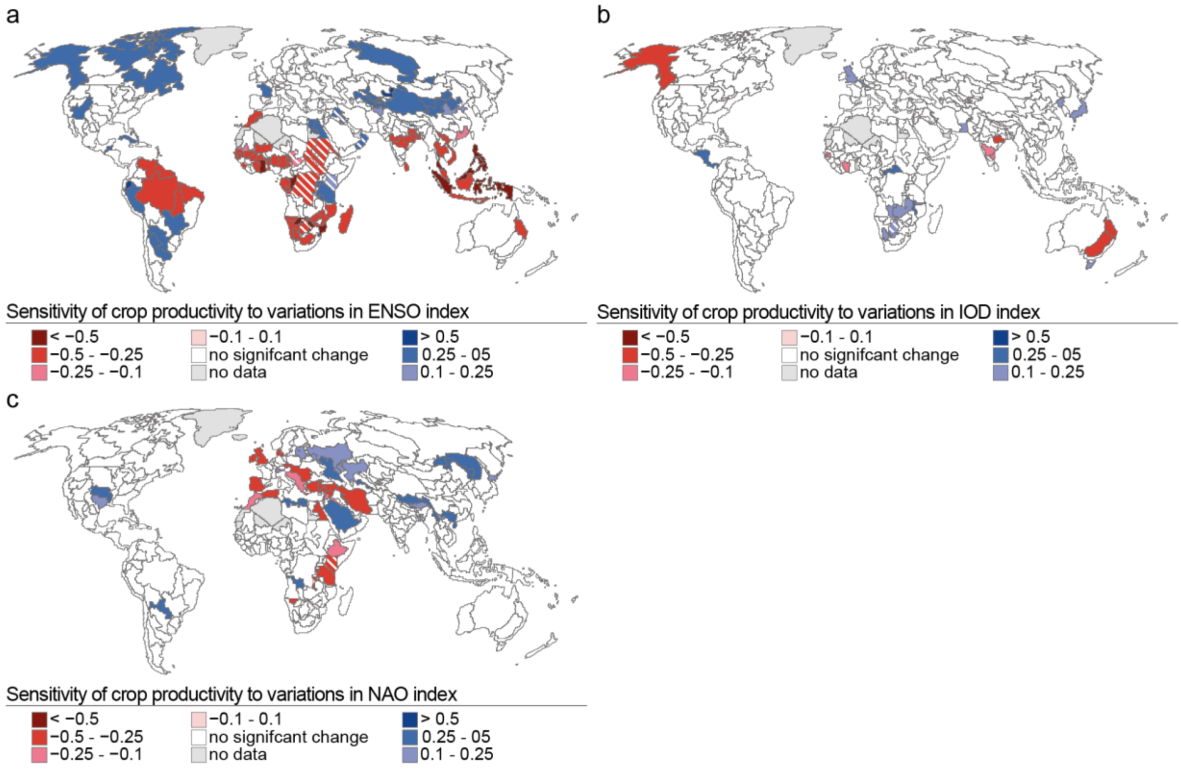

Supplementary Figure 23: Sensitivity of crop productivity (as derived from LPJmL model with limited irrigation input, see Methods) to changes in the a) ENSO, b) IOD and c) NAO indices at FPU level. The linear relationship was concluded to be significant based on the p-value (< 0.1, parametrized) of Pearson's correlation coefficient.

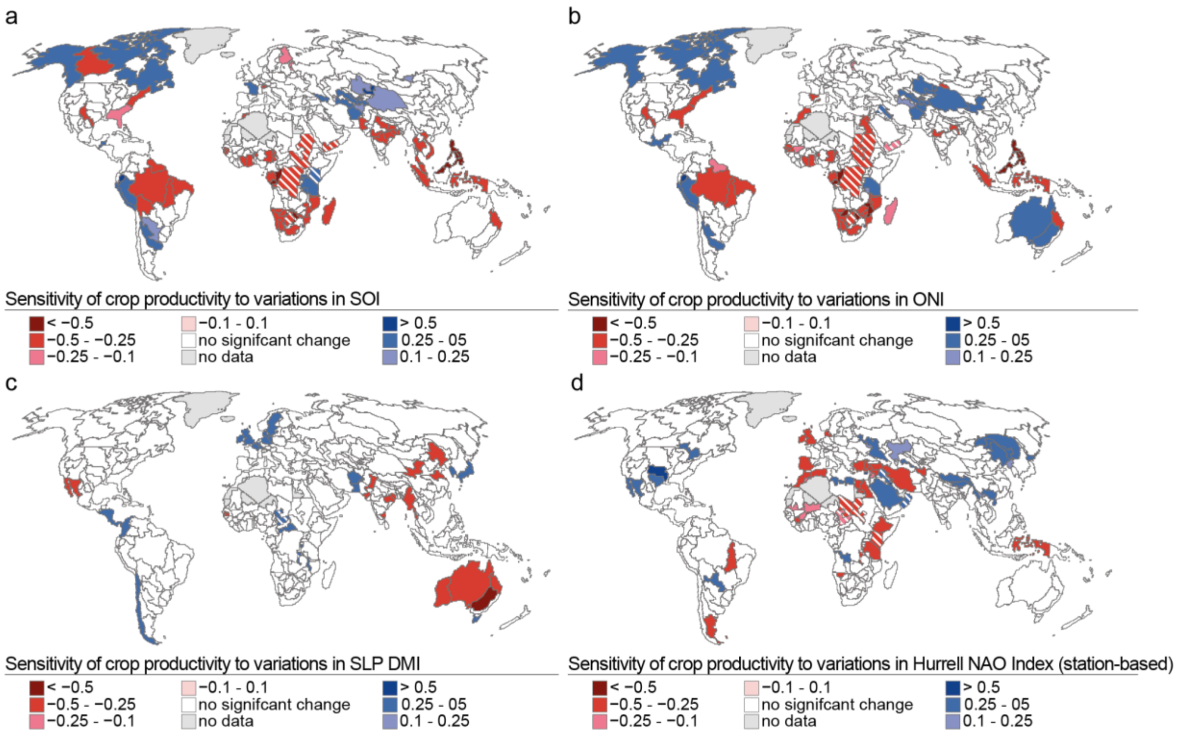

Supplementary Figure 24: Sensitivity of crop productivity to changes in a) the negative Southern Oscillation Index, b) the Oceanic Niño Index, c) the SLP Dipole Mode Index and d) the Hurrell North Atlantic Oscillation Index (station-based) at FPU level. The linear relationship was concluded to be significant based on the p-value (< 0.1, parametrized) of Pearson's correlation coefficient.

Supplementary Table 1: Regional changes in crop productivity during strongly oscillating years.

	**Change in crop productivity (%) during**					
**Region**	**Negative ENSO (La Niña)**	**Positive ENSO (El Niño)**	**Negative IOD**	**Positive IOD**	**Negative NAO**	**Positive NAO**
**Australia and Oceania**	-	-	6.0%	-8.0%	-	-
**Central America**	-	-	-	-	-	-
**East Asia**	-	-	-	-	–1.2%	-
**Eastern Europe and Central Asia**	-	-	-	-	-	-
**Middle East**	–4.1%	-	-	-	4.2%	–6.1%
**North Africa**	3.8%	-	-	-	5.8%	–5.8%
**North America**	-	-	-	–4.7%	-	-
**South America**	–4.3%	-	-	-	-	-
**South Asia**	1.9%	-	-	-	-	2.1%
**Southeast Asia**	2.8%	–1.4%	-	-	-	-
**Southern Africa**	1.7%	–3.6%	-	-	-	–2.6%
**Western Europe**	-	-	-	-	-	–1.8%

Supplementary Table 2: Regional Sensitivities of crop productivity to variations in the oscillation indices.

	Sensitivity of crop productivity to variations in		
**Region**	**ENSO index**	**IOD index**	**NAO index**
**Australia and Oceania**	-	–0.38	-
**Central America**	-	-	-
**East Asia**	-	-	0.29
**Eastern Europe and Central Asia**	-	-	-
**Middle East**	-	-	–0.41
**North Africa**	–0.25	-	–0.49
**North America**	-	-	-
**South America**	0.33	-	-
**South Asia**	–0.28	-	-
**Southeast Asia**	–0.53	-	-
**Southern Africa**	–0.51	-	-
**Western Europe**	-	-	-

Supplementary Table 3: Regional results for the coefficients of the multivariate regression analysis, describing the combined effects of the oscillations on crop productivity.

Region	ENSO Index	IOD Index	NAO Index	adjusted R^2
**Australia and Oceania**	0.217	–2.869***	0.865	0.222***
**Central America**	0.772*	–0.776	1.682#	0.099#
**East Asia**	0.846	–0.003	1.367*	0.084#
**Eastern Europe and Central Asia**	0.005	–0.019#	0.286	0.036
**Middle East**	–0.006	0.543	–1.259***	0.195**
**North Africa**	–2.598***	–0.236	–2.295***	0.394***
**North America**	–0.549	–0.778	1.115#	0.046
**South America**	1.843*	0.297	0.576	0.078#
**South Asia**	–0.327*	–1.153	0.799	0.116*
**Southeast Asia**	–2.835***	–0.156	–0.530	0.252***
**Southern Africa**	–2.440***	0.012	–0.840	0.307**
**Western Europe**	–0.012	1.180#	0.018#	0.116*

Statistical significance (N = 49):
# p < 0.1
* p < 0.05
** p < 0.01
*** p < 0.001

Supplementary Table 4: Population, cropland, and average total crop production (and percentage) in the areas where one or more oscillations produce significant changes in crop productivity during their negative and positive phases, when assessed at sub-national (i.e. FPU) scale. All areas included, also those where Pearson's correlation between reported and simulated crop productivity is insignificant ($p > 0.1$). Cf. Table 1 in main text.

**Oscillation**	**Population (10^9)**	**Cropland (10^6 km^2)**	**Crop production (10^{15} kcal yr^{-1})**
**ENSO**	1.7 (29%)	4.8 (31%)	2.4 (29%)
**IOD**	0.6 (11%)	2.0 (13%)	0.7 (9%)
**NAO**	2.3 (41%)	5.8 (37%)	3.4 (40%)
**Any**	**3.9 (69%)**	**10.7 (69%)**	**5.5 (66%)**

Supplementary Table 5: The indices chosen to represent the oscillations

Oscillation	Index name	Timespan	Index description	Conversion to yearly index values	Reference
ENSO	JMA SST Index	1868/03 - 2015/03	The index is calculated as a 5-month running mean of sea surface temperatures inside the 'Nino 3' region.	The mean of monthly index values from November (year N-1) to January (year N).	[8]
IOD	Dipole Mode Index	1958/01 - 2010/09	The dipole mode index is calculated as the normalized difference in SST anomaly between the tropical parts of western Indian Ocean and south-eastern Indian Ocean.	The mean of monthly index values from September (year N) to November (year N).	[5, 9]
NAO	Hurrell North Atlantic Oscillation (NAO) Index (PC-based)	1899/01 - 2015/04	The NAO index refers to the anomalies in the difference in normalized sea level pressure between the Azores and Iceland.	The mean of monthly index values from November (year N-1) to February (year N).	[7, 10]

Supplementary Table 6: Years when strong phases of ENSO, IOD and NAO were identified to have occurred.

ENSO, strongly oscillating years												
Negative	1965	1968	1971	1972	1974	1976	1985	1989	1996	1999	2000	2008
Positive	1966	1970	1973	1977	1983	1987	1988	1992	1998	2003	2007	2010

IOD, strongly oscillating years												
Negative	1964	1968	1970	1971	1974	1975	1980	1984	1992	1996	1998	2005
Positive	1961	1963	1967	1972	1976	1982	1987	1991	1994	1997	2002	2006

NAO, strongly oscillating years												
Negative	1962	1963	1964	1966	1969	1970	1977	1979	1996	2001	2006	2010
Positive	1973	1976	1983	1989	1990	1992	1993	1994	1995	2000	2007	2008

Supplementary Table 7: Cross-correlation (and p-values) for the explaining variables used in the multivariate regression calculations.

	**ENSO**	**IOD**	**NAO**
**ENSO**	1 (-)	–0.11 (0.43)	−0.050 (0.73)
**IOD**		1 (-)	0.099 (0.50)
**NAO**			1 (-)

Supplementary Table 8: VIF value of each variable used in the multivariate regression calculations.

	**VIF**
**ENSO**	1.01
**IOD**	1.02
**NAO**	1.01

Supplementary Table 9: Link functions used to linearize the crop productivity data for the multivariate regression analysis.

	**Linc Function**		
**FPU id**	**ENSO**	**IOD**	**NAO**
2	1 / x	cube root	1 / x
4	1 / x	power to 3	power to 2
5	-	square root	1 / x
7	-	square root	square root
8	exp(x)	log10	log10
9	-	power to 3	square root
10	power to 2	power to 3	log10
11	-	power to 3	cube root
12	power to 3	power to 3	power to 3
13	power to 3	cube root	power to 2
14	log	power to 2	1 / x
15	square root	1 / x	-
16	cube root	1 / x	1 / x
17	1 / x	power to 2	power to 3
18	log10	1 / x	1 / x
19	power to 2	1 / x	log10
20	power to 3	power to 3	log10
21	1 / x	power to 3	-
22	power to 2	1 / x	power to 2
23	power to 3	1 / x	square root
24	-	log10	exp(x)
25	cube root	1 / x	power to 2
26	-	log10	power to 3
27	1 / x	1 / x	power to 2
28	-	log10	power to 3
29	power to 3	power to 3	square root
30	power to 2	cube root	power to 2
31	log	power to 3	power to 3
32	power to 3	power to 3	-
33	cube root	power to 3	-
34	power to 3	power to 3	power to 3
35	log	-	power to 3
36	log	power to 2	power to 3
37	power to 3	log	square root
38	exp(x)	power to 3	square root
39	1 / x	power to 3	1 / x
40	1 / x	1 / x	power to 3
41	exp(x)	log10	1 / x
42	1 / x	-	log10
43	1 / x	1 / x	1 / x
44	cube root	power to 2	power to 3
45	exp(x)	log10	1 / x
46	square root	1 / x	power to 3
47	log	square root	power to 3
48	1 / x	power to 3	1 / x
49	-	square root	power to 3
50	square root	1 / x	1 / x

51	1 / x	power to 3	1 / x
52	log	log10	power to 3
53	1 / x	1 / x	cube root
54	power to 3	power to 3	cube root
55	log10	1 / x	-
56	power to 3	power to 3	square root
57	1 / x	cube root	1 / x
58	square root	1 / x	1 / x
59	square root	power to 3	1 / x
60	log10	power to 3	1 / x
61	1 / x	1 / x	log10
62	1 / x	power to 2	power to 2
63	power to 2	1 / x	power to 3
64	power to 3	log10	power to 2
65	power to 3	1 / x	cube root
66	cube root	1 / x	square root
67	log10	power to 3	1 / x
68	power to 3	log10	exp(x)
69	exp(x)	1 / x	power to 3
70	power to 3	-	power to 3
71	square root	power to 3	1 / x
72	cube root	-	power to 3
73	log	log10	power to 2
74	cube root	1 / x	power to 3
75	log	log10	cube root
76	power to 2	power to 3	power to 3
77	1 / x	1 / x	power to 3
78	power to 3	log10	log10
79	1 / x	cube root	power to 3
80	-	power to 3	exp(x)
81	square root	cube root	power to 3
82	1 / x	1 / x	power to 3
83	power to 2	power to 3	power to 3
84	1 / x	log10	power to 3
85	log	power to 3	power to 3
86	power to 3	1 / x	-
87	log	1 / x	power to 3
88	exp(x)	1 / x	power to 3
89	power to 3	log10	1 / x
90	-	1 / x	-
91	-	1 / x	power to 3
92	power to 2	-	log10
93	power to 3	log10	-
94	log	power to 3	square root
95	1 / x	power to 3	-
96	log10	log10	-
97	power to 2	1 / x	power to 2
98	log10	1 / x	cube root
99	log	square root	square root
100	1 / x	power to 2	power to 3

101	exp(x)	power to 2	-
102	1 / x	power to 3	1 / x
103	log	log10	1 / x
104	power to 3	power to 3	power to 3
105	1 / x	power to 3	power to 3
106	-	power to 2	power to 3
107	cube root	log10	power to 3
108	-	power to 2	log10
109	1 / x	square root	1 / x
110	square root	power to 3	1 / x
111	power to 3	1 / x	power to 3
112	-	1 / x	cube root
113	1 / x	cube root	power to 3
114	power to 3	1 / x	-
115	power to 3	1 / x	1 / x
116	exp(x)	power to 2	-
117	power to 3	1 / x	-
118	power to 3	1 / x	1 / x
119	square root	1 / x	log10
120	-	power to 3	power to 3
121	power to 2	1 / x	power to 3
122	exp(x)	power to 3	power to 3
123	-	power to 3	1 / x
124	power to 3	power to 3	1 / x
125	log10	1 / x	log10
126	square root	log10	1 / x
127	square root	power to 3	1 / x
128	-	1 / x	square root
129	-	exp(x)	power to 3
130	log	power to 2	square root
131	cube root	power to 3	1 / x
132	power to 2	power to 2	cube root
133	-	power to 2	power to 2
134	1 / x	power to 3	power to 2
135	power to 3	-	1 / x
136	1 / x	power to 2	cube root
137	square root	1 / x	log10
138	power to 3	1 / x	log10
139	power to 2	1 / x	1 / x
140	power to 3	-	log10
141	1 / x	square root	square root
142	power to 3	-	-
143	-	-	log10
144	power to 2	power to 2	power to 3
146	-	power to 3	power to 3
147	power to 3	1 / x	power to 3
148	power to 2	power to 3	log10
149	square root	square root	power to 2
150	1 / x	power to 3	power to 3

151	power to 2	log10	-
152	-	1 / x	power to 3
153	log10	1 / x	-
154	1 / x	log10	power to 2
155	-	1 / x	power to 3
156	1 / x	exp(x)	log10
157	cube root	square root	-
158	power to 3	power to 2	power to 3
160	log10	log10	-
162	1 / x	power to 2	power to 3
163	square root	power to 3	square root
164	exp(x)	square root	1 / x
165	-	1 / x	power to 3
166	power to 3	power to 2	power to 2
167	power to 2	power to 3	power to 3
168	1 / x	1 / x	log10
169	log	square root	square root
170	square root	log10	power to 3
171	power to 3	log10	power to 3
172	cube root	cube root	1 / x
173	cube root	1 / x	1 / x
174	1 / x	power to 3	log10
175	power to 2	square root	log10
176	1 / x	power to 3	log10
177	cube root	1 / x	log10
178	exp(x)	power to 3	-
179	1 / x	1 / x	power to 3
180	exp(x)	exp(x)	power to 2
181	exp(x)	power to 3	power to 3
182	-	power to 3	power to 3
183	1 / x	power to 3	1 / x
184	log10	power to 2	cube root
185	1 / x	cube root	1 / x
186	1 / x	power to 3	1 / x
187	log	power to 3	1 / x
188	1 / x	1 / x	cube root
189	1 / x	1 / x	1 / x
190	1 / x	log10	-
191	log10	-	cube root
192	log10	log10	square root
193	square root	log10	cube root
194	square root	1 / x	1 / x
196	power to 2	1 / x	power to 2
198	power to 3	cube root	power to 3
200	power to 3	1 / x	power to 3

201	cube root	power to 3	cube root
203	power to 3	power to 3	power to 3
204	1 / x	1 / x	log10
205	cube root	power to 3	1 / x
206	power to 3	-	log10
207	log	log10	1 / x
208	power to 3	power to 2	log10
209	exp(x)	power to 3	log10
210	exp(x)	power to 3	log10
211	cube root	power to 3	log10
212	exp(x)	power to 3	power to 3
213	-	1 / x	1 / x
214	square root	power to 3	exp(x)
215	1 / x	power to 2	power to 2
216	1 / x	power to 3	power to 3
217	1 / x	exp(x)	-
218	power to 2	-	power to 3
219	log	power to 3	-
220	exp(x)	power to 3	1 / x
221	log10	1 / x	1 / x
222	log10	log10	1 / x
223	log10	cube root	1 / x
224	square root	1 / x	power to 3
225	exp(x)	power to 3	log10
226	log	log10	power to 3
227	power to 3	power to 3	square root
228	log	log10	power to 3
229	1 / x	log10	power to 2
230	log10	1 / x	cube root
231	power to 2	log10	log10
232	1 / x	1 / x	exp(x)
233	1 / x	1 / x	power to 2
234	-	power to 3	cube root
235	cube root	1 / x	power to 3
236	log	1 / x	cube root
237	-	1 / x	1 / x
238	power to 2	1 / x	log10
239	power to 3	1 / x	power to 3
240	1 / x	1 / x	power to 3
242	power to 2	power to 3	1 / x
243	power to 2	cube root	1 / x
244	1 / x	log10	exp(x)
245	1 / x	log10	power to 2
246	power to 2	-	-
247	power to 3	1 / x	square root
248	power to 3	power to 2	-
249	power to 3	-	-
250	1 / x	power to 3	square root

251	square root	-	-
252	log10	-	log10
253	power to 3	log10	log10
254	-	1 / x	power to 3
255	1 / x	-	square root
256	log	-	1 / x
257	log	exp(x)	log10
258	exp(x)	log	square root
259	log	power to 2	1 / x
260	1 / x	square root	cube root
261	power to 3	power to 3	-
262	cube root	1 / x	1 / x
263	1 / x	log10	1 / x
264	log	log	power to 2
265	-	log10	1 / x
266	-	power to 3	power to 3
267	-	1 / x	1 / x
268	cube root	power to 2	cube root
269	1 / x	power to 3	1 / x
270	square root	square root	-
271	square root	square root	log10
272	1 / x	square root	power to 3
273	1 / x	-	power to 3
274	-	square root	power to 3
275	cube root	log10	power to 3
276	log	1 / x	log10
277	1 / x	-	-
278	-	-	1 / x
279	-	power to 3	-
280	log	1 / x	power to 3
281	1 / x	1 / x	power to 3
282	power to 3	-	1 / x
283	1 / x	power to 3	1 / x
284	log10	-	1 / x
285	log	1 / x	1 / x
286	log	-	power to 3
287	log	log10	power to 3
288	power to 3	power to 3	power to 3
289	power to 3	log10	cube root
290	-	-	square root
291	log	1 / x	log10
292	1 / x	log10	power to 2
293	-	1 / x	power to 3
294	1 / x	power to 3	square root
295	power to 3	1 / x	-
296	power to 2	square root	-
297	power to 2	power to 2	square root
298	power to 3	-	log10
299	square root	1 / x	cube root
300	square root	exp(x)	cube root

301	-	1 / x	log10
302	-	1 / x	-
303	square root	1 / x	power to 3
304	power to 3	-	cube root
305	1 / x	power to 3	-
306	square root	power to 3	1 / x
307	power to 3	1 / x	cube root
309	power to 3	power to 3	cube root

Supplementary Table 10. Global scale crop productivity calculations based on a) simulated and b) FAOSTAT[36] data. Please note that, here the simulated crop types do not directly correspond to the crop types of FAOSTAT. For example, wheat is simulated as a representative of temperate cereals, which also include barley, rye, and oat.

a: Global scale crop productivity calculated based on simulated data.

	**Average yield, (years 2001-2010, kg/yr)**	**Conversion factor (kcal/kg)**	**Average yield (years 2001 - 2010, 10^7 kcal/yr)**	**Harvested area, year 2000 (10^7 ha)**	**Production (10^{15} kcal)**
Cassava	12270	1090	1.34	1.67	0.22
Groundnuts	1798	4140	0.74	1.79	0.13
Maize	4823	3560	1.72	13.08	2.25
Millet	1139	3415	0.39	5.39	0.21
Pulses	829	3460	0.29	5.60	0.16
Rapeseed	1627	4940	0.80	2.17	0.17
Rice	4466	2800	1.25	10.21	1.28
Soybean	2360	3350	0.79	6.79	0.54
Sugar beet	47622	700	3.33	0.49	0.16
Sugarcane	61195	300	1.84	1.98	0.36
Sunflower seed	1307	3080	0.40	1.71	0.07
Wheat	3098	3283	1.02	24.14	2.46
			**Sum**	**75.03**	**8.01**
	**Harvested area weighted mean crop productivity (10^7 kcal/ha):**			**1.07**	

b: Global scale crop productivity calculated based on reported data (FAOSTAT[36]).

	**Yield (year 2010, kg/yr)**	**Conversion factor (kcal/kg)**	**Yield (year 2010, 10^7 kcal/yr)**	**Harvested area (year 2010, 10^7 ha)**	**Production (2010 10^{15} kcal)**
Cassava	12207	1090	1.33	1.97	0.26
Groundnuts, with shell	1662	4140	0.69	2.61	0.18
Maize	5190	3560	1.85	16.40	3.03
Millet	911	3400	0.31	3.60	0.11
Pulses, nes	744	3400	0.25	0.48	0.01
Rapeseed	1865	4940	0.92	3.21	0.30
Rice, paddy	4336	2800	1.21	16.17	1.96
Soybeans	2578	3350	0.86	10.28	0.89
Sugar beet	48638	700	3.40	0.47	0.16
Sugar cane	71075	300	2.13	2.37	0.50
Sunflower seed	1363	3080	0.42	2.31	0.10
Wheat	2971	3340	0.99	21.55	2.14
			**Sum**	**81.42**	**9.64**
	**Harvested area weighted mean crop productivity (10^7 kcal/ha):**			**1.18**	

Supplementary References

1. Trenberth, K. E. The definition of el nino. *Bull. Am. Meteorol. Soc.* **78**, 2771-2777 (1997).

2. Rasmusson, E. M. & Wallace, J. M. Meteorological aspects of the el nino/southern oscillation. *Science* **222**, 1195-1202 (1983).

3. Saji, N., Goswami, B. N., Vinayachandran, P. & Yamagata, T. A dipole mode in the tropical Indian Ocean. *Nature* **401**, 360-363 (1999).

4. Saji, N. & Yamagata, T. Possible impacts of Indian Ocean dipole mode events on global climate. *Climate Research* **25**, 151-169 (2003).

5. Hurrell, J. W. Decadal trends in the north atlantic oscillation: regional temperatures and precipitation. *Science* **269**, 676-679 (1995).

6. Hurrell, J. W., Kushnir, Y., Ottersen, G. & Visbeck, M. An overview of the North Atlantic oscillation. *Geophysical Monograph-American Geophysical Union* **134**, 1-36 (2003).

7. Ropelewski, C. F. & Halpert, M. S. Global and regional scale precipitation patterns associated with the El Niño/Southern Oscillation. *Mon. Weather Rev.* **115**, 1606-1626 (1987).

8. Trenberth, K. E. & Caron, J. M. The Southern Oscillation revisited: Sea level pressures, surface temperatures, and precipitation. *J. Clim.* **13**, 4358-4365 (2000).

9. Dai, A. & Wigley, T. Global patterns of ENSO-induced precipitation. *Geophys. Res. Lett.* **27**, 1283-1286 (2000).

10. Dai, A., Trenberth, K. E. & Karl, T. R. Global variations in droughts and wet spells: 1900-1995. *Geophys. Res. Lett.* **25**, 3367-3370 (1998).

11. Saji, N., Ambrizzi, T. & Ferraz, S. Indian Ocean Dipole mode events and austral surface air temperature anomalies. *Dyn. Atmos. Oceans* **39**, 87-101 (2005).

12. Ummenhofer, C. C. *et al*. What causes southeast Australia's worst droughts? *Geophys. Res. Lett.* **36** (2009).

13. Marchant, R., Mumbi, C., Behera, S. & Yamagata, T. The Indian Ocean dipole–the unsung driver of climatic variability in East Africa. *Afr. J. Ecol.* **45**, 4-16 (2007).

14. Ashok, K., Guan, Z. & Yamagata, T. Influence of the Indian Ocean Dipole on the Australian winter rainfall. *Geophys. Res. Lett.* **30** (2003).

15. Behera, S. K. & Yamagata, T. Subtropical SST dipole events in the southern Indian Ocean. *Geophys. Res. Lett.* **28**, 327-330 (2001).

16. Reason, C. Subtropical Indian Ocean SST dipole events and southern African rainfall. *Geophys. Res. Lett.* **28**, 2225-2227 (2001).

17. Hong, C., Lu, M. & Kanamitsu, M. Temporal and spatial characteristics of positive and negative Indian Ocean dipole with and without ENSO. *Journal of Geophysical Research: Atmospheres* **113** (2008).

18. Ashok, K., Guan, Z. & Yamagata, T. Impact of the Indian Ocean dipole on the relationship between the Indian monsoon rainfall and ENSO. *Geophys. Res. Lett.* **28**, 4499-4502 (2001).

19. Trigo, R. M., Osborn, T. J. & Corte-Real, J. M. The North Atlantic Oscillation influence on Europe: climate impacts and associated physical mechanisms. *Climate Research* **20**, 9-17 (2002).

20. Li, J., Yu, R. & Zhou, T. Teleconnection between NAO and climate downstream of the Tibetan Plateau. *J. Clim.* **21**, 4680-4690 (2008).

21. Cullen, H. M., Kaplan, A. & Arkin, P. A. Impact of the North Atlantic Oscillation on Middle Eastern climate and streamflow. *Clim. Change* **55**, 315-338 (2002).

22. Trigo, R. M. *et al*. North Atlantic Oscillation influence on precipitation, river flow and water resources in the Iberian Peninsula. *Int. J. Climatol.* **24**, 925-944 (2004).

23. McHugh, M. J. & Rogers, J. C. North Atlantic oscillation influence on precipitation variability around the southeast African convergence zone. *J. Clim.* **14**, 3631-3642 (2001).

24. Linderholm, H. W. *et al*. Interannual teleconnections between the summer North Atlantic Oscillation and the East Asian summer monsoon. *Journal of Geophysical Research: Atmospheres* **116** (2011).

25. Wang, B., Yang, J., Zhou, T. & Wang, B. Interdecadal changes in the major modes of Asian-Australian monsoon variability: strengthening relationship with ENSO since the late 1970s*. *J. Clim.* **21**, 1771-1789 (2008).

26. Cai, W., Cowan, T. & Sullivan, A. Recent unprecedented skewness towards positive Indian Ocean Dipole occurrences and its impact on Australian rainfall. *Geophys. Res. Lett.* **36** (2009).

27. Kripalani, R. & Kumar, P. Northeast monsoon rainfall variability over south peninsular India vis-à-vis the Indian Ocean dipole mode. *Int. J. Climatol.* **24**, 1267-1282 (2004).

28. Gershunov, A. & Barnett, T. P. Interdecadal modulation of ENSO teleconnections. *Bull. Am. Meteorol. Soc.* **79**, 2715-2725 (1998).

29. Li, J. *et al*. El Niño modulations over the past seven centuries. *Nature Climate Change* **3**, 822-826 (2013).

30. McPhaden, M. J., Zebiak, S. E. & Glantz, M. H. ENSO as an integrating concept in earth science. *Science* **314**, 1740-1745 (2006).

31. Cane, M. A. The evolution of El Niño, past and future. *Earth Planet. Sci. Lett.* **230**, 227-240 (2005).

32. Mann, M. E., Cane, M. A., Zebiak, S. E. & Clement, A. Volcanic and solar forcing of the tropical Pacific over the past 1000 years. *J. Clim.* **18**, 447-456 (2005).

33. Dettinger, M. D., Cayan, D. R., McCabe, G. J. & Marengo, J. A. in *Multiscale streamflow variability associated with El Nino/Southern oscillation* (Cambridge University Press, 2000).

34. McCABE, G. J. & Dettinger, M. D. Decadal variations in the strength of ENSO teleconnections with precipitation in the western United States. *Int. J. Climatol.* **19**, 1399-1410 (1999).

35. Ward, P. J., Eisner, S., Flörke, M., Dettinger, M. D. & Kummu, M. Annual flood sensitivities to El Niño–Southern Oscillation at the global scale. *Hydrology and Earth System Sciences* **18**, 47-66 (2014).

36. Food and Agriculture Organization of the United Nations (FAOSTAT) http://www.fao.org/faostat/en/#data/QC. Accessed on March 2017.

37. Willmott, C. J. Some comments on the evaluation of model performance. *Bull. Am. Meteorol. Soc.* **63**, 1309-1313 (1982).

38. Fader, M., Rost, S., Müller, C., Bondeau, A. & Gerten, D. Virtual water content of temperate cereals and maize: Present and potential future patterns. *Journal of Hydrology* **384**, 218-231 (2010).

39. Ray, D. K., Gerber, J. S., MacDonald, G. K. & West, P. C. Climate variation explains a third of global crop yield variability. *Nature communications* **6** (2015).
